# Bone marrow mesenchymal stem cell-derived small extracellular vesicles promote liver regeneration via miR-20a-5p/PTEN

**DOI:** 10.3389/fphar.2023.1168545

**Published:** 2023-05-25

**Authors:** Jing Zhang, Juan Gao, Xianlong Li, Dengna Lin, Zhihui Li, Jialei Wang, Junfeng Chen, Zhiliang Gao, Bingliang Lin

**Affiliations:** ^1^ Department of Infectious Diseases, The Third Affiliated Hospital of Sun Yat-sen University, Guangzhou, Guangdong, China; ^2^ Guangdong Key Laboratory of Liver Disease Research, The Third Affiliated Hospital of Sun Yat-sen University, Guangzhou, China; ^3^ Department of Anesthesiology, The Third Affiliated Hospital of Sun Yat-sen University, Guangzhou, Guangdong, China; ^4^ Key Laboratory of Tropical Disease Control (Sun Yat-sen University), Ministry of Education, Guangzhou, Guangdong, China

**Keywords:** acute liver failure, extracellular vesicles, liver regeneration, mesenchymal stem cells, miRNA

## Abstract

Balancing hepatocyte death and proliferation is key to non-transplantation treatments for acute liver failure (ALF), which has a high short-term mortality rate. Small extracellular vesicles (sEVs) may act as mediators in the repair of damaged liver tissue by mesenchymal stem cells (MSCs). We aimed to investigate the efficacy of human bone marrow MSC-derived sEVs (BMSC-sEVs) in treating mice with ALF and the molecular mechanisms involved in regulating hepatocyte proliferation and apoptosis. Small EVs and sEV-free BMSC concentrated medium were injected into mice with LPS/D-GalN-induced ALF to assess survival, changes in serology, liver pathology, and apoptosis and proliferation in different phases. The results were further verified *in vitro* in L-02 cells with hydrogen peroxide injury. BMSC-sEV-treated mice with ALF had higher 24 h survival rates and more significant reductions in liver injury than mice treated with sEV-free concentrated medium. BMSC-sEVs reduced hepatocyte apoptosis and promoted cell proliferation by upregulating miR-20a-5p, which targeted the PTEN/AKT signaling pathway. Additionally, BMSC-sEVs upregulated the mir-20a precursor in hepatocytes. The application of BMSC-sEVs showed a positive impact by preventing the development of ALF, and may serve as a promising strategy for promoting ALF liver regeneration. miR-20a-5p plays an important role in liver protection from ALF by BMSC-sEVs.

## 1 Introduction

Acute liver failure (ALF) has a high mortality rate, ranging from 50% to 80%, and is characterized by extensive short-term liver necrosis and apoptosis and failure to regenerate the liver. Viral hepatitis (hepatitis A, B, and E) and drug damage are the main causes of ALF (Bernal and McPhail, 2021). Orthotopic liver transplantation therapy can reduce mortality but is limited by the lack of donor organs and the high cost involved; less than 30% of ALF patients receive liver transplantation (Stravitz and Lee, 2019). Therefore, the treatment of ALF requires novel strategies to reduce hepatocyte death and promote liver regeneration.

Mesenchymal stem cell (MSC) therapy can improve liver function and increase survival in patients with liver failure (Li et al., 2016; Lin et al., 2017; Shi et al., 2012). In fact, MSC condined effect of the extracellular vesicles (EVs) and the proteins secreted by MSCs (Keshtkar et al., 2018; Wang et al., 2018). Small EVs (sEVs) are vesicles less than 200 nm in diameter that contain cytoplasmic proteins, RNAs, and lipids and are important for intercellular communication. The most widely studied sEVs are exosomes, which are derived from endosomes (Hade et al., 2021). In recent years, several studies have shown that MSC-sEVs of adipose and umbilical cord origin can treat acute liver injury (Liu et al., 2018; Song et al., 2021; Yan et al., 2017). However, the efficacy of human bone marrow MSC (hBMSC)-derived sEVs in the treatment of ALF is still unclear, and the key molecular mechanisms remain unknown.

Our previous study showed that miR-20a-5p expression was downregulated in primary hepatocyte exosomes of mice with carbon tetrachloride-induced liver failure and was upregulated by co-incubation with hBMSCs (Zhang et al., 2021). Current high-throughput data show that miR-20a-5p expression is downregulated in the liver tissues of hepatitis B virus-associated ALF patients (Diaz et al., 2015), and hBMSC-sEVs contain miR-20a-5p (Ferguson et al., 2018). Functional enrichment analysis of predicted target genes of miR-20a-5p showed that miR-20a-5p may regulate the AKT (also known as protein kinase B or PKB) pathway by targeting PTEN (phosphatase and tensin homolog deleted on chromosome ten), thereby regulating cell proliferation and apoptosis. It has been reported that BMSC transplantation promotes liver regeneration through the AKT/GSK3β (glycogen synthase kinase 3β) pathway (Ding et al., 2019). Therefore, we hypothesized that hBMSC-sEVs delivered miR-20a-5p to hepatocytes, thereby targeting PTEN to accelerate liver regeneration in ALF. In this study, we systematically evaluated the efficacy of hBMSC-sEVs in the treatment of lipopolysaccharide (LPS)/D-galactosamine (D-GalN)-induced ALF and investigated the regulatory and molecular mechanisms underlying the action of hBMSC-sEVs in hepatocyte apoptosis and proliferation *in vivo* and *in vitro*.

## 2 Materials and methods

### 2.1 Isolation and identification of BMSC-sEVs

Human BMSCs at passage three were provided by the Key Laboratory of Stem Cell and Tissue Engineering, Sun Yat-sen University. Isolation and purification were performed as previously described (Chen et al., 2018). BMSCs were cultured in animal-free medium and expanded to passage six. When the cells had grown to 80%–90% confluence, the cells were harvested using an Animal Component-Free Cell Dissociation Kit (Stemcell Technologies, Vancouver, Canada, #05426) and cultured at 1×10^4^ cells/cm^2^ in a 75 cm^2^ bottle using animal component-free medium (Stemcell Technologies, #05445). The proportion of viable cells for the cultures used for harvesting the sEVs and for the differentiation assays was above 95%.

At passage six, the surface expression of CD73 (#05811-80), CD90 (#03011-50), CD105 (#17111-80), CD34 (#06411-77), CD45 (#07111-77), HLA-DR (#74111-50), and isotype control FITC/PE/APC (#44212-50/77/80) was detected by a FACS Canto II flow cytometer (BD Biosciences, Franklin Lakes, NJ, United States). Antibodies were obtained from BioGems International Inc. (Westlake Village, CA, United States). Oil red O staining (ScienCell, #0843, Carlsbad, CA, United States) was performed after 2 weeks of culture with lipogenic differentiation medium (ScienCell, #7541) and Alizarin Red staining (ScienCell, #0223) was performed after 3 weeks of culture in osteogenic differentiation medium at passage six (ScienCell, #7531).

BMSC supernatants were collected every 48 h after adding fresh medium from passage three to six. As shown in Supplementary Figure S1, the dead cells were removed by centrifugation at 2000 *g* for 20 min at 4°C, and the cell debris was removed at 10,000 *g* for 30 min. Next, a 100 kD MWCO ultra-centrifugal filter (Merck Millipore, #UFC910096, Burlington, MA, United States) was used for 30-fold concentration. After centrifugation at 100,000 *g* for 70 min, the supernatant was collected and used as sEV-free CM. The precipitate was resuspended with PBS, centrifuged at 100,000 *g* for 70 min, and then resuspended again with PBS to obtain the sEVs. Both BMSC-sEVs and BMSC-CM^sEV-free^ were filtered and sterilized using 0.22 μm membrane filters (Merck Millipore, #SLHY033RB) and stored at −80°C.

BMSC-sEV identification was based on the Minimal Information for Studies of Extracellular Vesicles 2018 Guidelines (Thery et al., 2018). The cells, vesicles, and CM^sEV-free^ lysates (20 μg protein) were subjected to western blotting. The protein concentration was determined using the BCA method (KeyGen Biotech, #KGPBCA, Nanjing, People’s Republic of China). The expression of CD9 (25 kD, 1:1000, Abcam, #ab92726, Cambridge, United Kingdom), CD63 (37 kD, 1:1000, #ab134045), TSG101 (44 kD, 1:1000, #ab125011), and calnexin (68 kD, 1:1000, #ab133615) was detected. The concentrations and size distributions of BMSC-sEVs were assessed by nanoparticle tracking analysis using a Nanosight LM10 device (Malvern, Madrid, Spain) equipped with Nanosight NTA software 2.2 (Malvern). BMSC-sEV ultrastructures were observed under a H-7650 TEM transmission electron microscope at 80 kV (Hitachi, Tokyo, Japan). The results are shown in Supplementary Figure S2.

### 2.2 ALF murine model and treatment

C57BL/6 mice (specific pathogen free, 5-6 weeks of age, 18–22 g weight) were purchased from the Guangdong Medical Laboratory Animal Center. Animal experiments were approved by Laboratory Animal Ethics Committee (no. IACUC-G16012) and took place in SPF Animal Laboratory at Guangzhou Forevergen Biosciences. All experiments conformed to relevant regulatory standards. As shown in Supplementary Figure S3, mice were randomly divided into 13 groups: normal contrast (NC) group, ALF (Vehicle) 3/6/12/24 h group, BMSC-sEV-treated 3/6/12/24 h group, and BMSC-CM^sEV-free^-treated 3/6/12/24 h group. In the 3/6/12 h group, six mice per group were examined at the corresponding time points. In the NC/24 h group, 15 mice were used for monitoring survival, in which six were used for serum biochemical, western blot, qPCR, and liver histological analysis. Mice were administered 60 μg/kg LPS (Sigma, #L2880, St. Louis, MO, United States) and 800 mg/kg D-GalN (Gentihold, #cas:1772-03-8, Beijing, People’s Republic of China) by intraperitoneal injection to induce the ALF murine model. Next, immediate tail vein injections of PBS (200 μL), BMSC-sEVs (40 μg total protein/200 μL), or BMSC-CM^sEV-free^ (200 μL) were performed. Live mice were euthanized or dead mice were collected at 3/6/12/24 h after intraperitoneal injection, and mice of the NC group were euthanized at 24 h. For Kaplan–Meier survival rate analyses, the number of surviving mice in the normal and in 24 h groups were recorded per hour.

### 2.3 Serum biochemical and liver histological analysis

Serum and liver tissues of number 1–6 mice in each group were collected to assess the degree of liver injury. Serum aminotransferase (ALT) and aspartate aminotransferase (AST) levels were detected using the Roche Cobas 8000 automatic biochemical analyzer (Roche, Basel, Switzerland). Liver tissue was fixed in 4% paraformaldehyde (Biosharp, #BL539A, Hefei, People’s Republic of China) for over 24 h and embedded in paraffin blocks, sliced as 5-μm-thick sections, dewaxed with xylene, and dehydrated in 70% ethanol. Sections were stained with hematoxylin-eosin (H&E; Beyotime Biotechnology, #C0105, Shanghai, People’s Republic of China). Necrotic areas in H&E-stained sections were identified by the loss of cell structure, eosinophilic degeneration of the cytoplasm, pyknosis, karyolysis, and disappearance of nuclei. Necrotic areas on each slice were summed to calculate the ratio of the total area using ImageJ software.

Immunohistochemistry was used to detect the expression of proliferating cell nuclear antigen (PCNA). Liver tissue sections were de-paraffinized and rehydrated, followed by treatment with proteinase K (Merck Millipore, #1245680100) in a 37°C incubator to extract antigens. Tissue sections were incubated with a primary anti-PCNA antibody (1:100, #ab92552) and left overnight at 4°C. After multiple washes, tissue sections were labeled with horseradish peroxidase (HRP)-conjugated secondary antibody (1:500, #ab6721) for 1 h at 25°C to label the primary antibody. The sections were then counterstained with hematoxylin. After dehydration and fixation, the sections were observed under a light microscope. Nuclear positivity was calculated by the IHC profile plugin in ImageJ software. The intensity of positivity was evaluated as follows: negative, score 0; weak, score 1; moderate, score 2; and strong, score 3 (Vaz et al., 2020). The staining positivity was determined using the following formula: Overall Score = Positive Percentage Score × Intensity Score (Sun et al., 2016).

Apoptosis of liver tissue was detected using the TUNEL Apoptosis One Step Kit (red) (RiboBio Co. Ltd., #C11026, Guangzhou, People’s Republic of China) according to the manufacturer’s instructions. Positive rates were calculated by ImageJ. The values for each animal were calculated as the average in four randomly selected fields of view under a microscope.

### 2.4 Incubation of hepatocytes with BMSC-sEVs

The human normal hepatocyte cell line L-02 (Hu et al., 2013; Li et al., 2018) was purchased from Shanghai Zhong Qiao Xin Zhou Biotechnology Co., Ltd., and was verified using short tandem repeats (STR) analysis. Cell complete medium was prepared with RPMI 1640 (Gibco, #C22400500BT, Carlsbad, CA, United Statesa) + 10% FBS (Gibco, #10099141C) + 1% penicillin and streptomycin (Gibco, #15140122) for cell culture, and the medium was refreshed every 48 h. Cells were routinely passaged with trypsin (Gibco, #25200072).

The final concentration of BMSC-sEVs used was 15 μg/mL, and the same volume of PBS was added to the control group. Cell viability was measured using a CCK-8 assay (ApexBio, #K1018-JY, Houston, TX, United States). L-02 cells were seeded in 96-well plates at 2 × 10^3^/well, BMSC-sEVs were added when the cell density reached 30%–40%, and cells were cultured at 37°C and 5% CO2 for 48 h. CCK-8 was added at 0 h, 24 h, and 48 h, and OD values at 450 nm were measured 2 h later using a Flx800 spectrophotometer microplate reader (BioTEK Instruments, Winooski, VT, United States). The ALF model was established as previously described (Yan et al., 2017). L-02 cells were treated with 0.4 mM H2O2 (Aladdin, #Cas:7722-84-1, Shanghai, People’s Republic of China) when they reached 70%–80% confluence. After 6 h, the medium was replaced with fresh complete medium, and the culture was continued for 24 h. BMSC-sEVs were added and cell culture was continued for 24 h. CCK-8 solution was added at 0 h, 6 h, 24 h and 48 h, and OD values were measured 2 h later.

For detection of the cell cycle, apoptosis, and gene expression, L-02 cells were seeded at 2×10^5^/well in a 6-well plate. ALF cells were incubated with BMSC-sEVs for 24 h. The cell cycle was evaluated using PI staining, as previously reported (Lee et al., 2019). Cell apoptosis was determined by Annexin-V PI staining (BD Bioscience, #556547, Franklin Lakes, NJ, United States). Apoptosis rate = Q2% + Q3%. Cell proteins and RNAs were extracted from ALF cells after 24 h incubation with BMSC-sEVs.

### 2.5 BMSC-sEV tracking

DiR (Invitrogen, #D12731, Carlsbad, CA, United States) labeling of BMSC-sEVs was performed as previously reported (Wang et al., 2014). BMSC-sEVs were stained with 5 μM DiR for 15 min and washed three times with ExoQuick-TC (System Bioscience, #Exotc10a-1, Mountain View, CA, United States) exosome precipitation solution. DiR without sEVs was washed in the same way and resuspended with PBS as the control reagent. C57BL/6 male mice were randomly divided into three groups: the NC group, NC + BMSC-sEV group, and ALF + BMSC-sEV group (*n* = 3). Each mouse was injected with 40 μg DiR-labeled BMSC-sEVs or control reagent via the tail vein. ALF was then immediately induced. After 3 h and 6 h, the fluorescence distribution was measured using an *In-Vivo* FX Pro near-infrared imaging system (Bruker, Billerica, MA, United States). The mice were sacrificed at 6 h, and the liver, spleen, and kidney were collected to assess fluorescence intensities.

PKH26 labeling of BMSC-sEVs was performed as previously described (Chen et al., 2017). Briefly, BMSC-sEVs were stained with 4 μM PKH26 red dye (Sigma, #PKH26mini) for 15 min and the reaction was aborted with 10% exosome-free FBS (System Bioscience, #50A-1). The solution was washed three times with the precipitation method described above. PKH26 without BMSC-sEVs was used as a control reagent. L-02 cells were pre-seeded on 35 mm confocal Petri dishes (Nest, #801002, Jiangsu, People’s Republic of China), and ALF was induced by hydrogen peroxide. PKH26-labeled BMSC-sEVs (final concentration: 15 μg total protein/mL) or the same volume of control reagent were added and incubated for 3 h. The cells were fixed with 4% paraformaldehyde and washed with PBS. Actin was labeled with ActinGreen dye (GeneCopoeia, #C052T, Rockville, MD, United States), and nuclei were labeled with DAPI blue dye (Beyotime Biotechnology, #C1006). Images were taken using an LSM710 confocal microscope (Carl Zeiss, Oberkochen, Germany).

### 2.6 Next-generation sequencing of BMSC-sEV miRNA

BMSC-sEV RNA extraction, library construction, and miRNA sequencing were performed as described previously (Zhang et al., 2021). Clean reads were annotated using the miRBase (mature human miRNA database) to obtain known miRNAs. miRNA quantitation was performed using TPM (transcripts per million) analysis. The sequencing data have been deposited to the Sequence Read Archive repository (https://www.ncbi.nlm.nih.gov/sra), with the accession number PRJNA792027.

### 2.7 miRNA mimic/inhibitor transfection

The sequences of mimic NC, miR-20a-5p mimic, inhibitor NC, and miR-20a-5p inhibitor were designed and synthesized by GenePharma Co. (Suzhou, People’s Republic of China). Sequences are shown in Supplementary Table S1. Transfection was initiated at 30%–40% L-02 cell density, using a medium free of serum and antibiotics, riboFECT™ CP transfection kit (RiboBio Co. Ltd., #C10511), and mimic (100 nM)/inhibitor (200 nM), according to the manufacturer’s instructions. The medium was replaced with complete medium at 6 h after transfection and refreshed every 48 h thereafter. The viability of the normal L-02 cells was detected by CCK-8 at 0 h, 24 h, 48 h, and 72 h after transfection. ALF was induced at 24–48 h after transfection, when the cell density was above 70%. Cell viability was assessed at 0 h, 6 h, 24 h, 48 h, and 72 h after the addition of hydrogen peroxide. At 48 h after ALF induction, gene expression was measured, cell proliferation was detected by EdU staining (RiboBio Co. Ltd., #C10310), and apoptosis was detected by TUNEL staining (RiboBio Co. Ltd., #C11012), according to the manufacturer’s instructions. The positive rate of cells was calculated based on the average of four random fields per well under the microscope. The conditions of the rescue experiments were the same as those described above. Inhibitor transfection was performed in advance. BMSC-sEVs or the same volume of PBS were added 24 h after ALF induction. After 24 h incubation, EdU and TUNEL staining were performed and gene expression was detected.

### 2.8 RT-qPCR

Total RNA was extracted by TRIzol (Invitrogen, #15596026), and the RNA concentration and purity were determined by a NanoDrop One C spectrophotometer (Thermo Scientific, Waltham, MA, United States). Stem-loop RT primers were used for the reverse transcription of the miRNAs and hsa-mir-20a precursor. Total RNA (1 μg) was reverse-transcribed using a kit (Takara, #RR047A, Dalian, People’s Republic of China). The cDNA was amplified using a qPCR kit (Takara, #RR820A). U6 served as an internal reference. Also, 2 μg of the mRNAs and mmu-mir-20a precursor were reverse-transcribed using Transcriptor Universal cDNA Master (Roche, #5893151001) and qPCR was performed using SYBR Green I Master (Roche, #4887352001). GAPDH was used as an internal reference. Reverse transcription was performed using a 2720 Thermal Cycler DNA thermal cycler (ABI, Foster City, CA, United States). qPCR was performed using the Roche LightCyCler480 under the StepOnePlus Real-Time PCR system (Applied Biosystems, Foster City, CA, United States). All procedures were performed following the reagent vendor’s instructions. hsa-mir-20a precursor and U6 primer were purchased from RiboBio Co. Ltd. (#MQPS0000803-1; #MQPS0000002-1). The remaining primer sequences are shown in Supplementary Table S2. The 2^−ΔΔCT^ method was used for data analysis of qRT-PCR experiments (Moroney et al., 2020).

### 2.9 Western blot

According to the manufacturer’s instructions, cells and homogenized liver tissues were dissolved in a lysate buffer containing protease inhibitors and phosphorylation inhibitors (KeyGen Biotech, #KGP250). The solution was mixed with loading buffer (Fude Biotechnology, #FD002, Hangzhou, People’s Republic of China) and denatured at 100°C for 10 min. About 20 μg of protein from each group was separated on a 10% (w/v) sodium dodecyl sulfate-polyacrylamide gel electrophoresis gel (Epizyme, #PG113, Shanghai, People’s Republic of China) and transferred to a 0.22/0.45-μm polyvinylidene fluoride membrane (Merck Millipore, #ISEQ00010/#IPVH00010). The membrane was blocked with 5% skim milk (BD, #232100) or 5% BSA (Sigma, #B2064) for 1 h, washed with TBST, and incubated overnight with primary antibodies at 4°C, including PTEN (55 kD, 1:1000, ab170941), AKT (56 kD, 1:10,000, ab179463), S473pAKT (56 kD, 1:5000, ab81283), GSK3β (46 kD, 1:5000, ab32391), S9pGSK3β (47 kD, 1:1000, ab75814), cleaved caspase-3 (19 kD, 1:1000, Cell Signaling Technology, CST#9664, Danvers, MA, United States), and GAPDH (37 kD, 1:10,000, ab3602) antibodies. The membrane was then washed three times with TBS. The membrane was incubated with secondary antibodies (HRP; Goat anti-rabbit IgG, 1:2000, ab6702) at room temperature for 1 h. Again, the membrane was washed three times with TBS. The bands were visualized using the Ecl Plus kit (Beyotime Biotechnology, #P0018) and Fluorchem M Imager (ProteinSimple, San Jose, CA, United States). ImageJ software (National Institutes of Health, Bethesda, MD, United States) was used for the quantitative analysis of gray images.

### 2.10 Target gene prediction, KEGG enrichment, and network analysis

The target genes of miRNA were predicted with the use of DIANA TOOLS (miTG score > 0.7) (https://diana.imis.athena-innovation.gr/DianaTools). Prediction of the binding site of miRNA and transcription was done by TargetScanHuman 7.2 (https://www.targetscan.org/). Kyoto Encyclopedia of Genes and Genomes (KEGG) pathway enrichment and network analyses and visualization were performed using the online OmicShare cloud platform (https://www.omicshare.com/tools/).

### 2.11 Statistical analysis

Data analysis was performed, and graphics were created using GraphPad Prism 8 and Adobe Illustrator^®^ CC 2019. Survival analysis was performed using log-rank (Mantel-Cox) analysis. Comparison of multiple conditions was done with one or two-way ANOVA. Differences in gene expression were assessed using the two sample Student’s *t*-test when normality assumption was met. Otherwise, the Mann-Whitney *U* test was used. The Shapiro-Wilk test was performed to evaluate the normality assumption for all samples. Six independent experimental replicates were used *in vivo*. At least three independent experiments were performed *in vitro*. Data are presented as mean ± SEM. Statistical significance was set at *p* < 0.05 (Segovia et al., 2019).

## 3 Results

### 3.1 BMSC-sEVs improve prognosis of LPS/D-GalN-induced ALF, attenuate apoptosis and necrosis of liver cells, and promote cell proliferation

**FIGURE 1:**
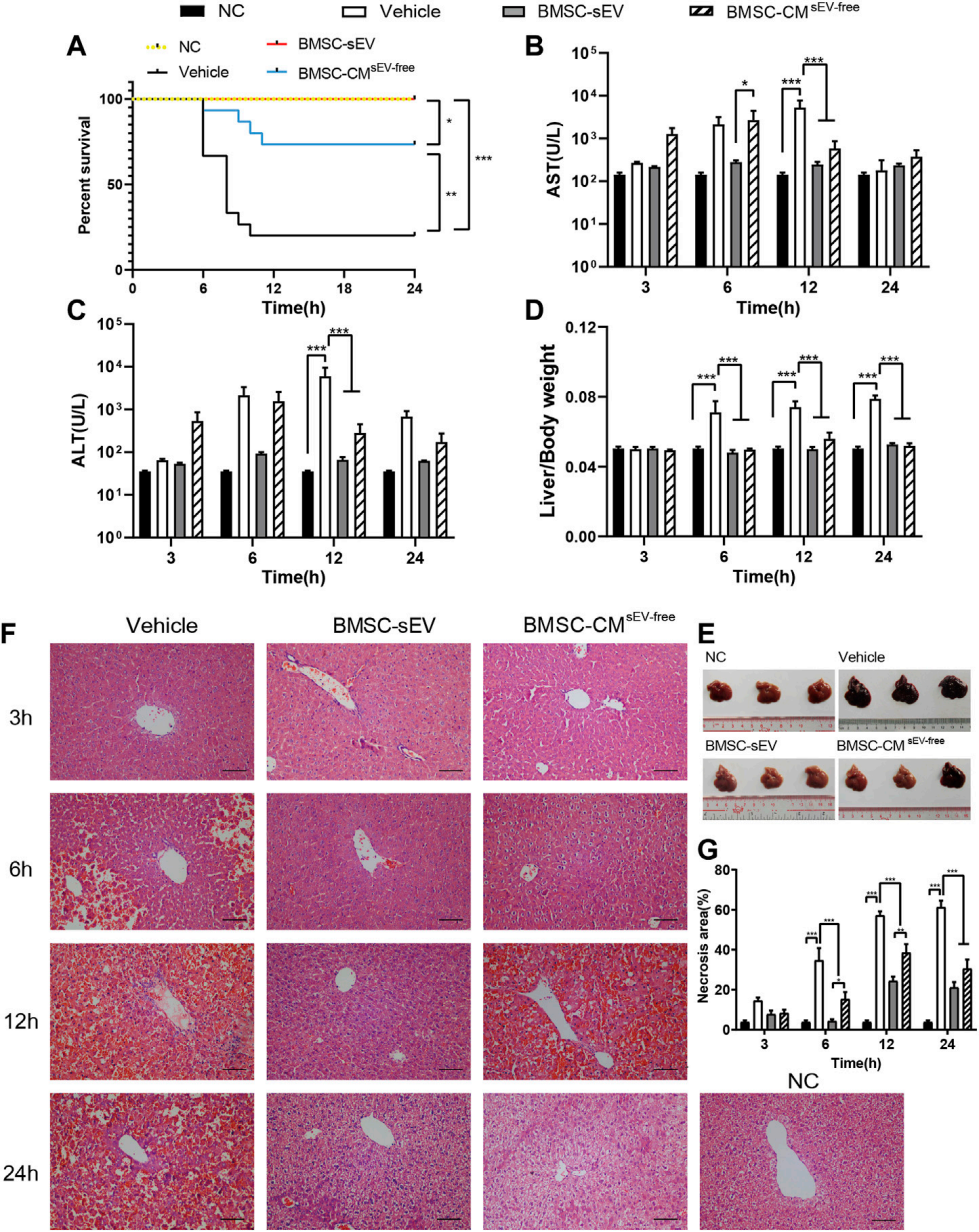
BMSC-sEVs exert a significant preventive effect on LPS/D-GalN-induced acute liver failure. **(A)** BMSC-sEVs were more effective than BMSC-CM^sEV-free^ in terms of improving 24-h survival. **(B–D)** At 12 h, serum levels of ALT and AST and liver/body weights were elevated by LPS/D-GalN injection (Vehicle group) and were significantly decreased by treatment with BMSC-sEVs or BMSC-CM^sEV-free^. **(E)** Liver tissues after 12 h of treatment. Three out of six samples are shown. **(F)** H&E staining of liver tissues. Scale bar: 100 μm. **(G)** Necrosis rates of liver tissues. For each animal, the value was calculated from the average of four randomly chosen fields of view under the microscope. Data are presented as mean ± SEM. Statistical analysis of survival was performed using log-rank (Mantel-Cox) test, *n* = 15; others were assessed by two-way ANOVA, *n* = 6. *p < 0.05, **p < 0.01, ***p < 0.001.

In our study, the 24 h survival rate of LPS/D-GalN-induced ALF mice was 20%. No death was observed in mice treated with BMSC-sEVs, while the 24 h survival rate of the BMSC-CM^sEV-free^ (concentrated BMSC conditioned medium without sEV) group was 73.3%. BMSC-sEV was significantly more effective in improving 24-h survival than BMSC-CM^sEV-free^ was (*p* = 0.04). In addition, the deaths of ALF mice mainly occurred 6–12 h after LPS/D-GalN induction (Figure 1A).

In ALF mice, serum ALT and AST levels were significantly increased at 12 h (vs. NC group, both *p* < 0.001), while both sEV and CM^sEV-free^ treatment significantly decreased ALT and AST levels (vs. Vehicle group, all *p* < 0.001). At 6 h, the sEV treatment significantly reduced AST levels compared with those in the CM^sEV-free^ group (*p* = 0.04) (Figures 1B, C). BMSC-sEV or CM^sEV-free^ treatment reduced the liver/body weight ratio, liver congestion, and liver swelling after 6 h (Figures 1D, E). H&E staining showed that ALF mice developed typical features of liver necrosis, including collapse of cell structure, eosinophilic degeneration of the cytoplasm, pyknosis, karyolysis, and the disappearance of nuclei. Compared with that of the Vehicle group, the hepatocyte necrosis rate of the sEV/CM^sEV-free^ group decreased significantly after 6 h (6, 12, 24 h, *p* < 0.001). In addition, liver necrosis in the sEV group was notably reduced compared with that in the CM^sEV-free^ group (6 h, *p* = 0.04, 12 h, *p* = 0.004) (Figures 1F, G). Although both treatments showed positive effects relating to the prevention of ALF development, sEV treatment showed better therapeutic efficacy than CM^sEV-free^ treatment did.

**FIGURE 2:**
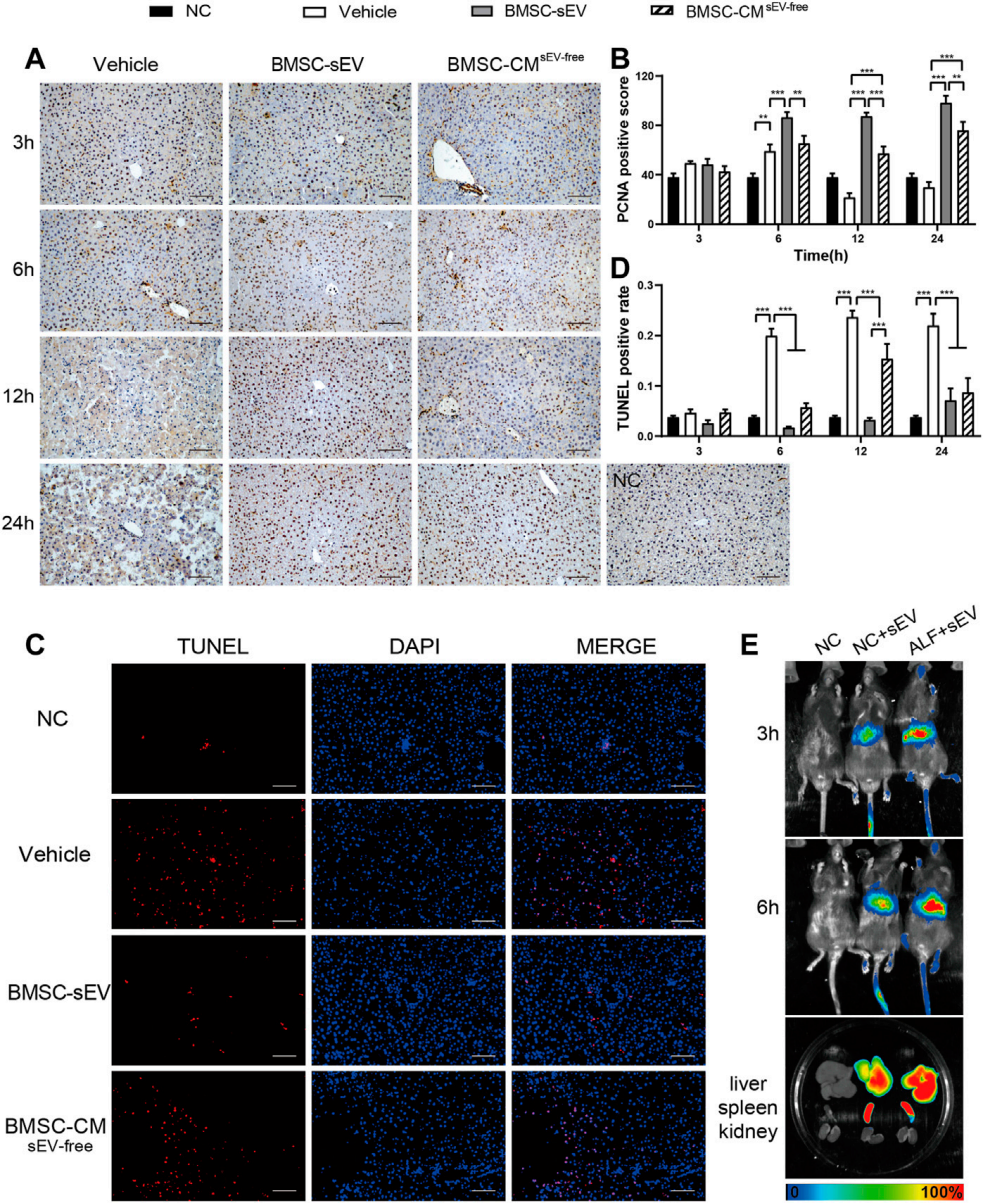
BMSC-sEV infusion enhances liver regeneration in LPS/D-GalN-induced acute liver failure. **(A)** Representative images of PCNA (dark brown nuclei) staining in liver tissue. Scale bar: 100 μm. **(B)** PCNA-reactive hepatocyte nuclei were quantified by digital image analysis. **(C)** Representative images of TUNEL staining in liver tissue at 12 h. Scale bar: 100 μm. **(D)** Quantification of TUNEL-positive cells. **(E)** Distribution of DiR-labeled BMSC-sEVs in organs at 3/6 h after intravenous injection in mice. Data are presented as mean ± SEM. *n* = 6. For each animal, the value was calculated from the average of four randomly chosen fields of view under the microscope. Statistical analysis was performed using two-way ANOVA, *p < 0.05, **p < 0.01, ***p < 0.001.

Proliferation and apoptosis of hepatic parenchymal cells are important factors affecting the prognosis of ALF. As shown in Figures 2A, B, proliferating cell nuclear antigen (PCNA) immunostaining revealed that sEV and CM^sEV-free^ treatment significantly promoted liver cell proliferation in ALF mice at 12 h and 24 h (vs. Vehicle, all *p* < 0.001). At 12 h, BMSC-sEV displayed a more pronounced pro-proliferation profile (vs. CM^sEV-free^ group, *p* < 0.001). As presented in Figures 2C, D and Supplementary Figure S4, the terminal deoxynucleotidyl transferase (TdT) dUTP nick-end labeling (TUNEL) staining showed that the apoptosis of liver cells in the sEV or the CM^sEV-free^ group was decreased after 6 h (vs. Vehicle, *p* < 0.001); moreover, apoptosis in the sEV group was notably lower than that in the CM^sEV-free^ group at 12 h (*p* < 0.001).

*In vivo* imaging analysis showed that DiR-labeled BMSC-sEVs were mainly localized in the livers of mice at 3 h after injection through the tail vein, and BMSC-sEV recruitment was more obvious in the livers of mice with ALF. BMSC-sEVs were significantly aggregated in the liver and spleen at 6 h (Figure 2E).

### 3.2 BMSC-sEVs regulate miR-20a-5p/PTEN in the liver of ALF mice

**FIGURE 3:**
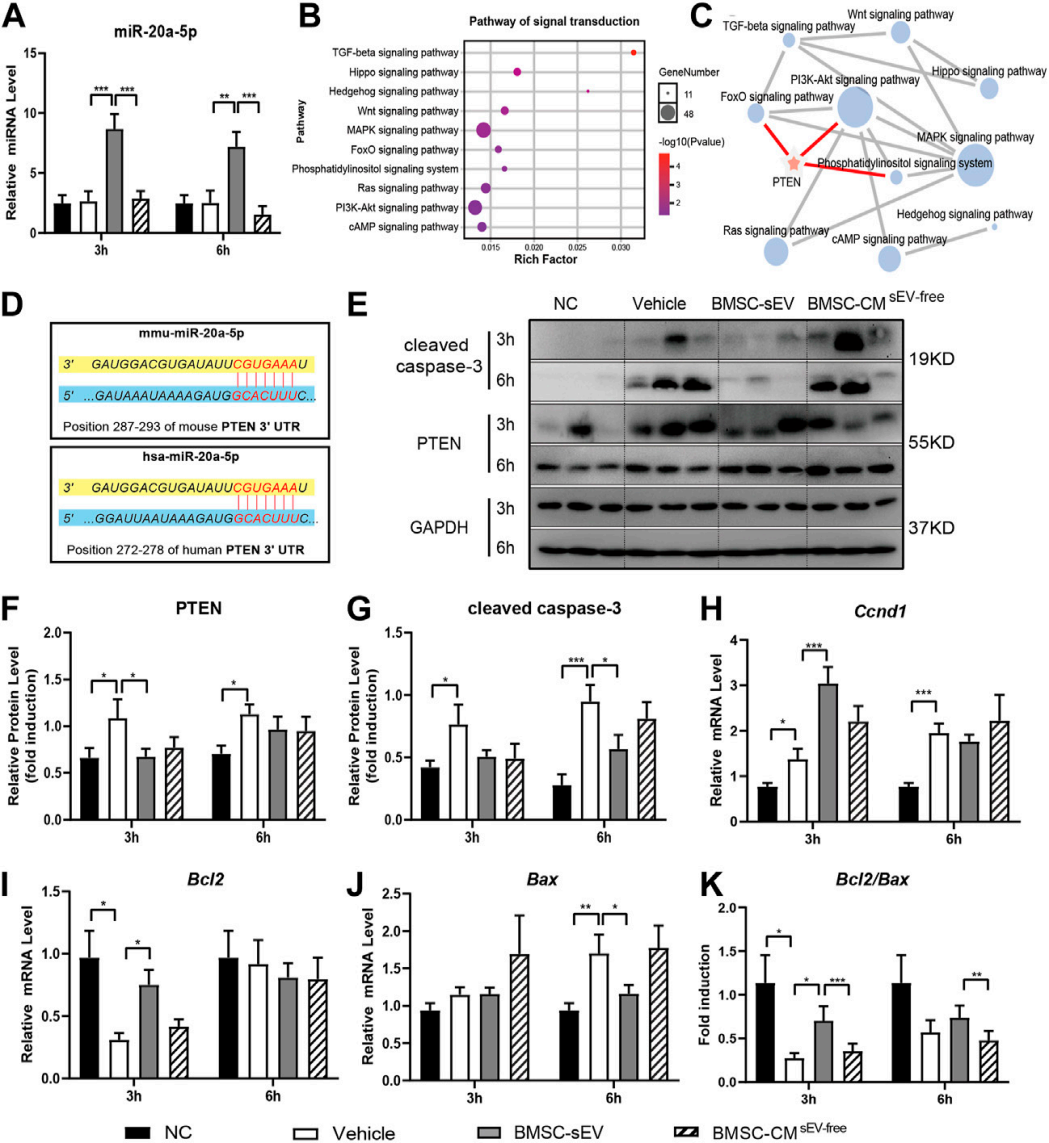
miR-20a-5p is upregulated by BMSC-sEVs in the livers of ALF mice, and could target PTEN. **(A)** The expression levels of miR-20a-5p in liver tissues of mice with LPS/D-GalN-induced ALF after BMSC-sEV treatment or BMSC-CM^sEV-free^ therapy. **(B)** KEGG analysis showed that the target genes of hsa-miR-20a-5p were significantly enriched into 10 pathways related to signal transduction. **(C)** Pathway network analysis showed that the PI3K/AKT pathway was an important regulatory target of miR-20a-5p, through targeting PTEN. **(D)** Predicted sequence pairing of the PTEN 3′ UTR region (bottom) and miR-20a-5p (top). Protein levels of PTEN and cleaved caspase-3 were determined by **(E)** western blotting and then **(F-G)** quantified by assessing the gray values. **(H–J)** qPCR was used to detect the relative expression levels of *Ccnd1*, *Bcl2*, and *Bax* in liver tissues, and **(K)** the *Bcl2/Bax* ratio was calculated. Data are presented as mean ± SEM. Statistical analysis was performed using Student’s *t*-test, *p < 0.05, **p < 0.01, ***p < 0.001, *n* = 6.

In ALF mice, BMSC-sEV treatment induced a remarkable increase in the levels of liver miR-20a-5p (Figure 3A). KEGG enrichment analysis showed that the target genes of hsa-miR-20a-5p were associated with ten pathways related to signal transduction (*p* < 0.05; Figure 3B). Further network analysis revealed that the PI3K/AKT pathway, which is related to proliferation and apoptosis, may be key, and the target gene PTEN is involved in the regulation of this pathway (Figure 3C). The seed sequence of miR-20a-5p was fully complementary to the 3’ UTR segment of PTEN at the 7mer-m8 site, in both human and mouse sequences (Figure 3D). For mice in the BMSC-sEV group, liver PTEN expression was decreased relative to that in the Vehicle group (3 h; Figures 3E, F). Meanwhile, *CCND1* and *BCL2* expression was obviously upregulated (3 h; Figures 3H, I). In contrast, *BAX* (6 h; Figure 3J) and cleaved caspase-3 expression (6 h; Figures 3E, G) was shown to be downregulated. *BCL2/BAX* ratios were found to be increased (3 h; Figure 3K). Compared with that in the Vehicle group, there was no significant change in the expression of these genes in the BMSC-CM^sEV-free^ group.

**FIGURE 4:**
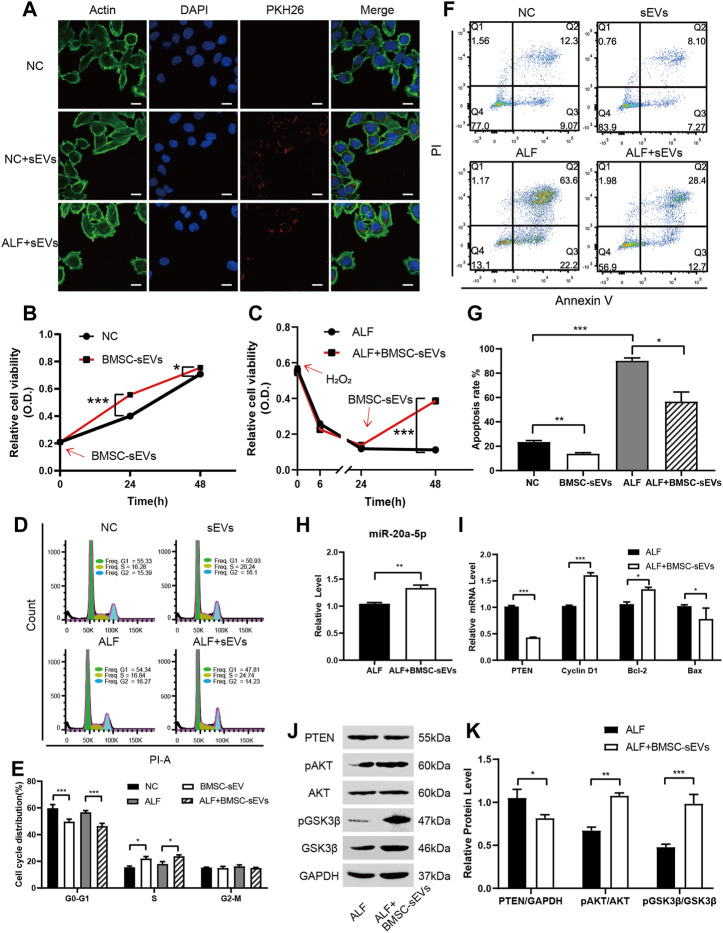
BMSC-sEVs promote hepatocyte proliferation and G1/S transformation and inhibit apoptosis *in vitro* via miR-20a-5p/PTEN/AKT. **(A)** Confocal microscopy revealed that PKH26-labeled BMSC-sEVs were located in the actin-positive cytoplasm region of L-02 cells. Scale bar: 25 μm. **(B)** Normal or **(C)** injured L-02 cells were incubated with BMSC-sEVs and cell viability was detected using the CCK-8 assay. **(D-E)** PI staining was used to evaluate the cell cycle and **(F-G)** Annexin V-PI staining was used to detect apoptosis after 24 h of incubation. **(H)** qPCR was used to detect the relative expression levels of miR-20a-5p, *PTEN, CCND1, BCL2*, and BAX **(I)**. **(J-K)** Western blotting was used to detect PTEN, pAKT, AKT, pGSK3β, and GSK3β protein levels. Data are presented as mean ± SEM. Statistical analysis was performed using Student’s *t-*test, *p < 0.05, **p < 0.01, ***p < 0.001, *n* = 3.

*In vitro*, BMSC-sEVs labeled with the red dye PKH26 were co-incubated with either normal L-02 cells or L-02 cells with hydrogen peroxide-induced acute injury for 3 h. As observed by confocal microscopy, BMSC-sEVs were distributed in the cytoplasm and clustered around the nucleus. This confirmed that BMSC-sEVs could be internalized by normal and injured liver cells (Figure 4A). A Cell Counting Kit-8 (CCK-8) assay showed that the proliferation rates of normal and injured hepatocytes increased significantly after co-incubation with BMSC-sEVs for 24 h (Figures 4B, C). Propidium iodide (PI) staining showed a decrease in the number of cells in the G1 phase and an increase in the number of cells in the S phase of the cell cycle (Figures 4D, E). Annexin-V-PI double staining indicated decreased apoptosis of hepatocytes (Figures 4F, G). We observed that 24 h co-incubation with BMSC-sEVs upregulated miR-20a-5p expression (Figure 4H), reduced PTEN expression, and promoted AKT and GSK3β phosphorylation in L-02 cells with hydrogen peroxide-induced injury (Figures 4J, K). Meanwhile, the expression of *CCND1* and *BCL2* was upregulated and that of *BAX* was downregulated (Figure 4I).

### 3.3 miR-20a-5p inhibits hepatocyte apoptosis and promotes proliferation via the PTEN/AKT pathway

**FIGURE 5:**
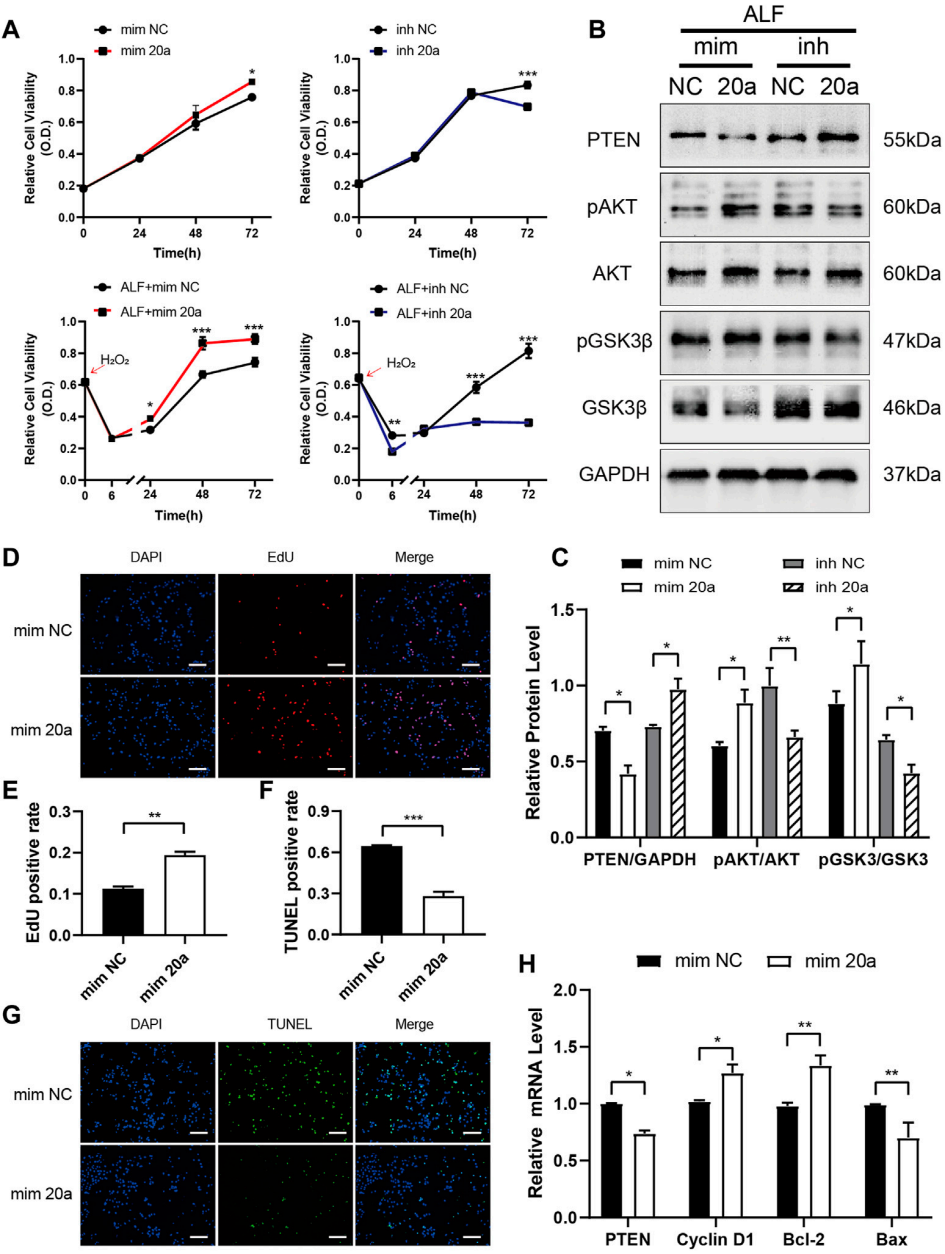
miR-20a-5p promotes hepatocyte proliferation and inhibits apoptosis via the PTEN/AKT pathway. **(A)** The viability of L-02 cells transfected with miR-20a-5p mimic/inhibitor was tested using the CCK-8 assay under normal conditions or after hydrogen peroxide-induced damage. **(B-C)** Western blotting was used to detect protein levels at 48 h after hydrogen peroxide-induced damage in L-02 cells transfected with miR-20a-5p mimic/inhibitor. **(D-E)** EdU staining was used to detect proliferation at 48 h after hydrogen peroxide-induced damage in L-02 cells transfected with miR-20a-5p mimic. **(F-G)** TUNEL staining was used to detect apoptosis. Scale bar: 200 μm. The positive rate of cells was calculated based on the average of four random fields per well under the microscope. **(H)** qPCR was used to detect the relative expression levels of *PTEN, CCND1, BCL2*, and *BAX*. Data are presented as mean ± SEM. Statistical analysis was performed using Student’s *t-*test, *p < 0.05, **p < 0.01, ***p < 0.001. *n* = 3.

In normal L-02 cells or those with hydrogen peroxide-induced acute injury, the transfection of miR-20a-5p mimic increased hepatocyte viability, whereas the transfection of miR-20a-5p inhibitor decreased cell viability (Figure 5A). Upregulation of miR-20a-5p expression inhibited PTEN expression and promoted AKT and GSK3β phosphorylation in injured L-02 cells, whereas downregulation of miR-20a-5p expression had the opposite effect (Figures 5B, C). In injured L-02 cells, the upregulation of miR-20a-5p expression promoted proliferation (Figures 5D, E), inhibited apoptosis (Figures 5F, G), and upregulated *CCND1* and *BCL2* and downregulated *PTEN* and *BAX* expression at the transcriptional level (Figure 5H).

### 3.4 BMSC-sEVs promote hepatocyte proliferation in a miR-20a-5p dependent manner

**FIGURE 6:**
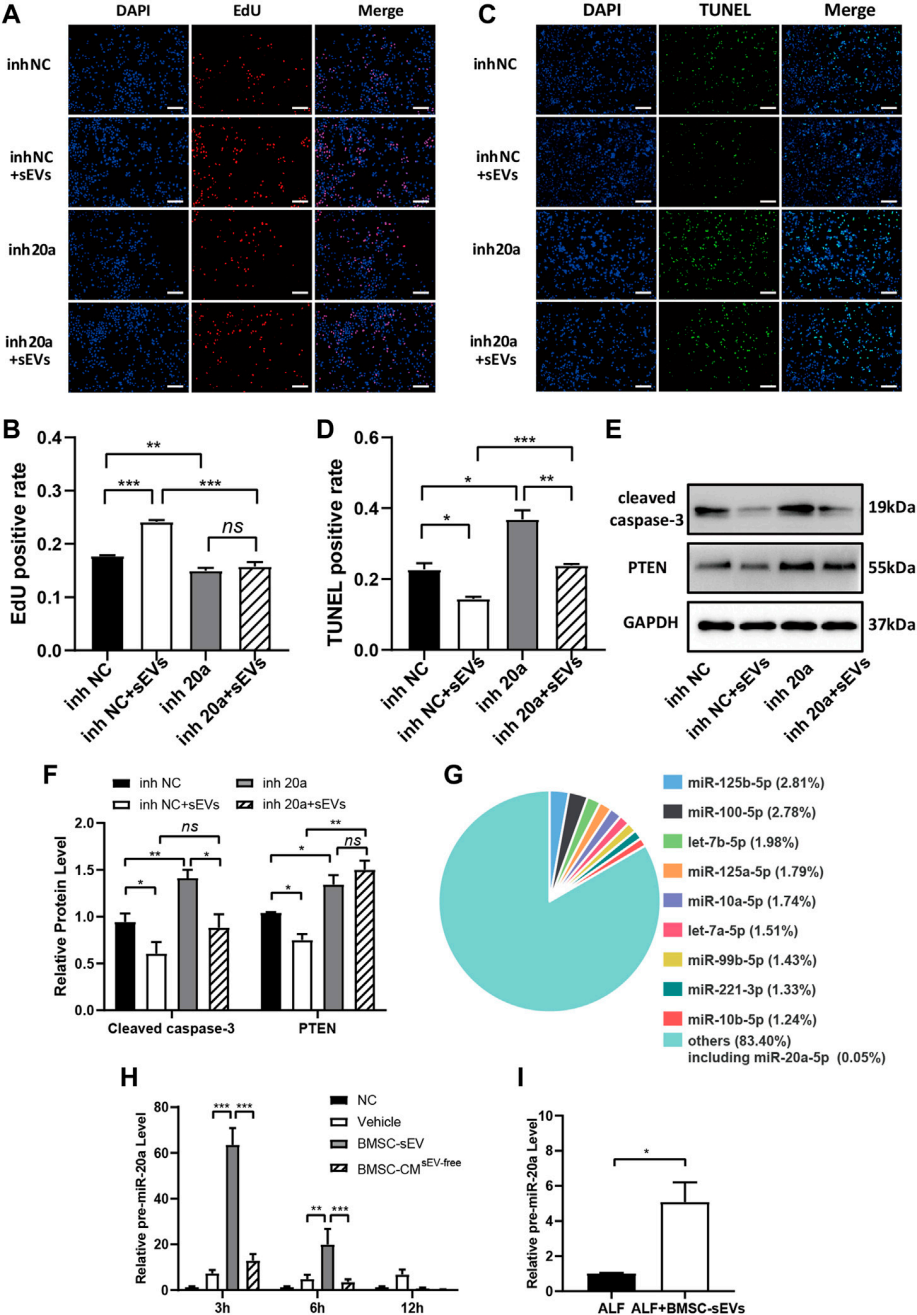
BMSC-sEVs inhibit PTEN expression and promote proliferation through miR-20a-5p but inhibit apoptosis independently of miR-20a-5p. L-02 cells transfected with miR-20a-5p inhibitors were rescued with BMSC-sEVs after hydrogen peroxide-induced injury. **(A-B)** EdU staining was used to detect cell proliferation. **(C-D)** Detection of apoptosis by TUNEL staining. Scale bar: 200 μm. The positive rate of cells was calculated based on the average of four random fields per well under the microscope. *n* = 3. **(E-F)** Protein levels of PTEN and cleaved caspase-3. *n* = 3. **(G)** Levels of miR-20a precursors in liver tissues of mice with LPS/D-GalN-induced ALF after BMSC-sEV treatment. *n* = 6. **(H)** The intracellular miR-20a precursor levels were detected at 24 h after adding BMSC-sEVs to hydrogen peroxide-damaged L-02 cells. *n* = 3. Data are presented as mean ± SEM. Statistical analysis was performed using Student’s *t-*test, *p < 0.05, **p < 0.01, ***p < 0.001.

BMSC-sEV treatment of injured L-02 cells transfected with miR-20a-5p inhibitor did not result in significant changes to the positive rate of EdU relative to the inhibitor NC group (Figures 6A, B). Although TUNEL-positive rates were increased (Figures 6C, D), cleaved caspase-3 levels (Figures 6E, F) were significantly reduced. In addition, BMSC-sEVs could not inhibit PTEN expression after transfection with miR-20a-5p inhibitor (Figures 6E, F). Therefore, the inhibition of miR-20a-5p could block the regulation of PTEN as well as the pro-proliferative effect of BMSC-sEVs on injured L-02 cells but could not completely block the anti-apoptotic effect.

Next, we investigated whether BMSC-sEVs upregulated miR-20a-5p expression in hepatocytes by direct transmission only. miRNA sequencing results of hBMSC-sEVs indicated that the relative abundance of miR-20a-5p was 0.05% (Figure 6G, Supplementary Table S3). Further experiments showed that at the early stage of BMSC-sEV treatment in mice with ALF, there was a transient upregulation of levels of the precursor miR-20a in the liver (Figure 6H). Moreover, miR-20a precursor levels were upregulated in injured L-02 cells after 24 h incubation with BMSC-sEVs (Figure 6I).

## 4 Discussion

MSCs contribute to tissue repair and have been used for liver regeneration (Alfaifi et al., 2018; Lee et al., 2018; Wang et al., 2018). However, factors such as the low survival rate of transplanted cells, potential vascular embolism, potential immunogenicity, and carcinogenic risk have limited the clinical application of MSCs (Driscoll and Patel, 2019). sEV therapy is advantageous over cell therapy owing to the nanoscale volumes, lower amounts of membrane proteins, lack of a complete genome, and ease of production and storage. Therefore, sEVs are considered an ideal alternative to traditional MSC therapies (Lou et al., 2017). sEVs secreted from MSCs of different tissue sources have been found to have various beneficial effects, such as modulating innate immunity, attenuating apoptosis and ferroptosis, promoting proliferation, preventing fibrosis, and suppressing oxidative stress (Borrelli et al., 2018; Lin et al., 2022; Tian et al., 2022; Zhao et al., 2022). In previous reports, BMSC-derived sEVs were suggested to be involved in the direct regulation of hepatocyte survival. Human and mouse BMSC-derived sEVs were found to attenuate liver injury in mice with lethal liver failure by attenuating hepatocyte apoptosis (Haga et al., 2017). Exosomes secreted by BMSCs alleviated LPS/D-GalN-induced apoptosis of primary rat hepatocytes *in vitro* (Zhao et al., 2019). In addition, hBMSC-derived EVs promoted the proliferation of liver cells in the late stage of hepatic ischemia reperfusion injury in rats (Anger et al., 2019). In this study, we identified a novel mechanism through which hBMSC-sEVs promote the maintenance of the hepatocyte pool in ALF, which is closely related to the prognosis. To our knowledge, the present study shows, for the first time, that hBMSC-sEVs play a protective role against ALF by upregulating miR-20a-5p and downregulating PTEN expression, reducing apoptosis, and promoting the proliferation of hepatocytes.

MSCs promote the repair of injured tissue mainly through paracrine action. In 2008, van Poll et al. reported that hBMSC-CM (25-fold concentrated, >3 kD) reduced hepatocyte apoptosis and promoted proliferation in ALF rats (van Poll et al., 2008). Later, Damania et al. found, *in vitro* and *in vivo*, that BMSC-EVs alleviated liver injury more effectively than rat BMSC-CM with the same protein content did (Damania et al., 2018). Monguio-Tortajada et al. compared the effects on T cells of umbilical cord MSC EVs, CM without EV fraction, and complete CM and suggested that EVs were the main bioactive component of MSCs responsible for inhibiting T cell activity (Monguio-Tortajada et al., 2017). However, the protein content of the secretome obtained from CM was notably higher than that from sEVs. Moreover, as the secretome composition is complex, it is necessary to study the fractionated secretome and use higher therapeutic doses. Our research showed, for the first time, that BMSC-sEVs significantly reduced liver damage in mice with ALF induced by LPS/D-GalN, attenuating hepatocyte apoptosis more significantly and promoting proliferation earlier than 30-fold concentrated BMSC-CM (components greater than 100 kD) did. This may be because sEVs accumulate in the liver more quickly than secreted proteins. BMSC-sEVs naturally aggregate to the liver, and several studies have shown that MSC-sEVs are mainly distributed in the liver and spleen in mice (Chen et al., 2017; Wang et al., 2014; Zheng et al., 2020). Second, similar to the mechanism of MSC migration to the injury site (Maxson et al., 2012), chemokine receptors from the mother cell plasma membrane on sEV surfaces promote the migration of sEVs to the inflammatory site. Concentrating the conditioned medium substantially increases the dose of protein that can be administered for treatment; however, the removed low-molecular-weight proteins contain certain active ingredients, and the efficacies of sEVs and these low-molecular-weight proteins have not yet been compared. The decrease in TUNEL positive rate in the sEV group was accompanied by downregulation of cleaved caspase-3 expression, indicating that sEVs inhibit apoptosis in the ALF liver. Despite the decreased TUNEL positive rate in the CM^sEV-free^ group, no significant downregulation of cleaved caspase-3 expression was observed, indicating that CM^sEV-free^ may inhibit hepatic necrosis by reducing necroptosis or other non-apoptotic cell death pathways than apoptosis.

**FIGURE 7:**
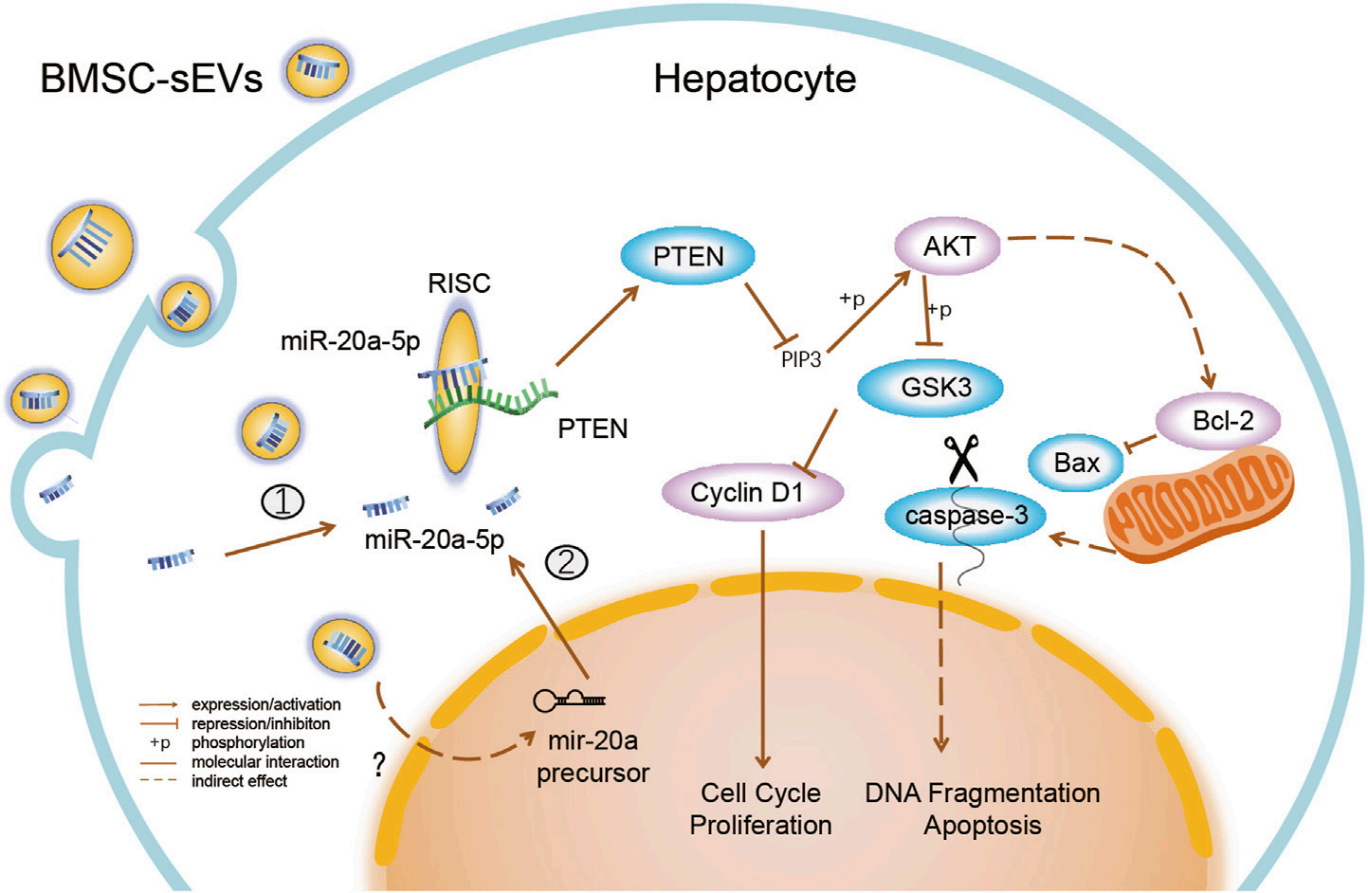
BMSC-sEVs regulate miR-20a-5p/PTEN in hepatocytes in ALF. BMSC-sEVs fuse with hepatocyte membranes or are degraded after endocytosis, and may bring mature miR-20a-5p into cells ①. BMSC-sEVs promote the increase in levels of intracellular miR-20a-5p precursors through an unknown mechanism and then promote the endogenous synthesis of miR-20a-5p in hepatocytes ②. Upregulated miR-20a-5p targets the 3′ UTR of transcripts and assists in the RNA-induced silencing complex (RISC) to degrade transcripts and inhibit PTEN translation. Downregulation of PTEN expression promotes cell proliferation and inhibits apoptosis by activating AKT and regulating downstream molecules.

MSCs secrete cytokines such as prostaglandin E2, indoleamine 2,3-dioxygenase (IDO), and soluble human leukocyte antigen G5 (HLA-G5), which alleviate liver injury by inhibiting overactivated immune responses (Moroney et al., 2020; Volarevic et al., 2014). Here, we identified the mechanism by which MSC-derived sEVs directly promoted hepatocyte regeneration. BMSC-sEVs could migrate quickly to the damaged tissue after peripheral intravenous infusion and were taken up by liver cells within a short period of time. Subsequently, BMSC-sEVs promoted the upregulation of miR-20a-5p levels and inhibited PTEN expression in the livers of mice with ALF. Several studies have demonstrated, through luciferase reporter assays, that miR-20a-5p directly targets the PTEN 3’ UTR in endothelial cells and some tumor cells (Gao et al., 2019; Tian et al., 2020; Zhang et al., 2015). *In vitro*, we verified that both BMSC-sEVs and miR-20a-5p regulated cell proliferation and apoptosis through the PTEN/AKT pathway. These results suggest that BMSC-sEVs promote hepatocyte proliferation and inhibit apoptosis and explain the observed therapeutic effects of BMSC-sEVs on ALF (Figure 7).

miR-20a-5p is closely involved in liver pathophysiology. Previous studies have shown that the downregulation of liver miR-20a-5p expression is associated with inflammation and fibrosis (Fu et al., 2020), and that the upregulation of miR-20a-5p expression reduces excessive lipid accumulation (Wang et al., 2020) and promotes liver glycogen synthesis (Fang et al., 2016). Therefore, miR-20a-5p is thought to be a key mediator in the mechanism by which BMSC-sEVs promote GSK3β Ser9 inactivating phosphorylation, which leads to the activation of glycogen synthase. Zhang et al. reported that exosomes containing miR-20a in umbilical cord MSCs alleviated liver ischemia/reperfusion (I/R) injury, possibly because miR-20a downregulated Beclin-I and Fas expression in the livers of I/R rats, thereby suppressing liver cell apoptosis (Zhang et al., 2020). Clearly, miR-20a is a key molecule that aids MSC-sEVs in promoting the repair of acute liver injury. From the results of our analysis and other studies, the relative abundance of miR-20a-5p in hBMSC-sEVs was lower (0.05%–0.22%) (Ferguson et al., 2018). Therefore, it can be inferred that the induction of miR-20a-5p synthesis may be a more important factor in the upregulation of miR-20a-5p in hepatocytes by hBMSC-sEVs than the direct delivery of mature miR-20a-5p. However, the exact underlying mechanisms are unclear. BMSC-sEVs may also contribute to the synthesis of mature miR-20a-5p by transporting precursors. Interestingly, our previous study revealed that the level of exosomal miR-20a-5p in the hepatic microenvironment was elevated after mice with liver failure were exposed to MSCs (Zhang et al., 2021). We thus speculate that hepatocytes produce miR-20a-5p-rich second-generation sEVs, which exert domino-like regulation for the generation of distant injured hepatocytes. Another unexpected finding was that the ability of BMSC-sEVs to promote hepatocyte proliferation relied on miR-20a-5p, but the ability to inhibit apoptosis did not. It has been reported that BMSC-sEVs can attenuate apoptosis by promoting autophagy (Borrelli et al., 2018) and inhibiting oxidative stress (Liao et al., 2019). Therefore, further investigation of alternative mechanisms by which sEVs participate in the regulation of ALF hepatocyte apoptosis is necessary.

One clinical concern is that BMSC-sEV-mediated regulation of miR-20a-5p/PTEN might lead to the unrestricted proliferation of hepatocytes. In this study, the expression of the precursor and mature miR-20a-5p in the livers of mice treated with BMSC-sEVs peaked at 3 h and then decreased gradually. In fact, sEVs that are taken up can be degraded by lysosomes or re-released through early/sorting endosomes (Pegtel and Gould, 2019; Kalluri and LeBleu, 2020) and hence these effects may not be stable in the long term. In addition, we found that in the injured L-02 cells, MYC expression was downregulated after 24 h incubation with BMSC-sEVs (Supplementary Figure S5). This is a negative feedback loop, as the transcription factor c-Myc also binds the pri-miR-17-92a transcriptional E-box element and activates the promoter (O’Donnell et al., 2005). In conclusion, the pro-proliferation signal transmitted by BMSC-sEVs is strictly controlled via the signaling crosstalk.

However, many questions remain to be addressed in order to realize practical application. First, it is unclear whether BMSC-sEVs loaded with miR-20a-5p can achieve greater benefits than unmodified sEVs or miR-20a-5p agomir for the treatment of ALF. Second, the long-term efficacy and safety of modified/unmodified BMSC-sEV therapy need to be observed. The optimal dose, duration, and route of administration of BMSC-sEVs need to be further explored. During *in vitro* culture, human BMSC has higher expanding ability in speed and yield compared to mouse BMSC. It can be speculated that more sEVs may be harvested from human-derived BMSCs with the same number of primary BMSCs in the same culture period. Although the use of mouse BMSC may better mimic the actual effects of BMSC on injured hepatocytes in mice, using human BMSC could highlight the conserved mechanisms of action across species, and the engineered sEV constructed based on these mechanisms may be more readily validated by models of different species origins. Human-derived MSC-sEV is more economical, ethical, safe, and has more potential as a clinical therapy to treat human diseases.

## 5 Conclusion

This study suggests that BMSC-sEVs can treat LPS/GalN-induced ALF by inhibiting hepatocyte apoptosis and promoting proliferation. BMSC-sEV-mediated regulation of miR-20a-5p/PTEN may be key to promote liver repair in ALF. This study provides theoretical support for the application of BMSC-sEVs in the treatment of ALF, novel insights into the mechanisms underlying BMSC-sEV-accelerated liver regeneration in ALF, and a new intervention target for future translational studies.

## Data Availability

The datasets presented in this study can be found in online repositories. The names of the repository/repositories and accession number(s) can be found in the article/Supplementary Material.
