# Supplementary material for: Bone marrow mesenchymal stem cell-derived small extracellular vesicles promote liver regeneration via miR-20a-5p/PTEN
Source: Front Pharmacol. 2023 May 25;14:1168545. doi: 10.3389/fphar.2023.1168545 (PMC10248071; doi:10.3389/fphar.2023.1168545)
Supplement: Supplementary file 2 [file Table1.DOCX]

Table.S1 Transfection sequences

| Name | Sequence (5′–3′) | Length |
| --- | --- | --- |
| mimic NC | Sense UUCUCCGAACGUGUCACGUTT  Antisense ACGUGACACGUUCGGAGAATT | 21  21 |
| miR-20a-5p mimic | Sense UAAAGUGCUUAUAGUGCAGGUAG  Antisense ACCUGCACUAUAAGCACUUUAUU | 23  23 |
| inhibitor NC | CAGUACUUUUGUGUAGUACAA | 21 |
| miR-20a-5p inhibitor | CUACCUGCACUAUAAGCACUUUA | 23 |

Table.S2 Primer sequences for RT-qPCR

| Gene | Specie | Primer | Sequence (5′–3′) | Length |
| --- | --- | --- | --- | --- |
| miR-20a-5p | hsa/mmu | sl | GGTTGTTGGTTGGTTGGTTGTATCCAACAACCCTACCT | 38 |
|  |  | F | TGCGTAAAGTGCTTATAGTGC | 21 |
|  |  | R | GTTGTTGGTTGGTTGGTTGT | 20 |
| miR-10a-5p | hsa | sl | GGCTGTTGTGTTGTGTTGTGGATACAACAGCCCACAAA | 38 |
|  |  | F | GGGTACCCTGTAGATCCGAA | 20 |
|  |  | R | GCTGTTGTGTTGTGTTGTGG | 20 |
| miR-22-3p | hsa | sl | GTCCTCCTCTCCTCTCCTCTCATGAGGAGGACACAGTT | 38 |
|  |  | F | GGGAAGCTGCCAGTTGAAG | 19 |
|  |  | R | TCCTCCTCTCCTCTCCTCTC | 20 |
| miR-26b-5p | hsa | sl | GGTGTGGTGTGGTATGGTGTGATCACCACACCACCTAT | 38 |
|  |  | F | AGGGCGTTCAAGTAATTCAGG | 21 |
|  |  | R | GTGTGGTGTGGTATGGTGTG | 20 |
| miR-29a-3p | hsa | sl | GTCCTCCTCTCCTCTCCTCTCATGAGGAGGACTAACCG | 38 |
|  |  | F | AGGGGTAGCACCATCTGAAAT | 21 |
|  |  | R | TCCTCCTCTCCTCTCCTCTC | 20 |
| miR-148a-3p | hsa | sl | GGAGAGGAGAGGAAGAGGGAAATCTCCTCTCCACAAAG | 38 |
|  |  | F | GGGTCAGTGCACTACAGAA | 19 |
|  |  | R | GAGAGGAGAGGAAGAGGGAA | 20 |
| miR-363-3p | hsa | sl | GTCCTCCTCTCCTTCCTTCTCATGAGGAGGACTACAGA | 38 |
|  |  | F | GGGAATTGCACGGTATCCA | 19 |
|  |  | R | TCCTCCTCTCCTTCCTTCTC | 20 |
| U6 | mmu | sl | TCGTATCCATGGCAGGGTCCGAGGTATTCGCCATGGATACGACACAAAAATATGGAACGCTT | 62 |
|  |  | F | GTGCTCGCTTCGGCAGCACA | 20 |
|  |  | R | TGGCAGGGTCCGAGGT | 16 |
| mir-20a pre | mmu | F | CCTGCGTGGTGTGTGTGAT | 19 |
|  |  | R | GGCGAGGCTGGAGTTCTAC | 19 |
| PTEN | hsa | F | AGGGCTTCAATTTCACTTCTT | 21 |
|  |  | R | TTGTACTCCGCTTAAAATCGT | 21 |
| Cyclin D1 | hsa | F | GCGGAGGAGAACAAACAG | 18 |
|  |  | R | CACAGAGGGCAACGAAG | 17 |
| BCL2 | hsa | F | TTCATCGTCCCCTCTCC | 17 |
|  |  | R | TCAGTCCGGTATTCGCA | 17 |
| BAX | hsa | F | TGCGTCCACCAAGAAGC | 17 |
|  |  | R | TCCAGTTCGTCCCCGAT | 17 |
| GAPDH | hsa | F | CCTTCCGTGTCCCCACT | 17 |
|  |  | R | GCCTGCTTCACCACCTTC | 18 |
| Cyclin D1 | mmu | F | ACCCTGACACCAATCTCCT | 19 |
|  |  | R | CTCCTTCTGCACGCACTT | 18 |
| BCL2 | mmu | F | AAACCCTCCATCCTGTCC | 18 |
|  |  | R | TCCTAAACCCTGCTTCCC | 18 |
| BAX | mmu | F | TGCGTCCACCAAGAAGC | 17 |
|  |  | R | CCACCCGGAAGAAGACC | 17 |
| GAPDH | mmu | F | TGTTTCCTCGTCCCGTAGA | 19 |
|  |  | R | ATCTCCACTTTGCCACTGC | 19 |

RT-qPCR, Reverse transcription quantitative polymerase chain reaction; hsa, human; mmu, mouse; pre，precursor; sl, stem loop; F, forward; R, reverse.
