# Supplementary material for: Bone marrow mesenchymal stem cell-derived small extracellular vesicles promote liver regeneration via miR-20a-5p/PTEN
Source: Front Pharmacol. 2023 May 25;14:1168545. doi: 10.3389/fphar.2023.1168545 (PMC10248071; doi:10.3389/fphar.2023.1168545)
Supplement: Supplementary file 3 [file DataSheet1.docx]

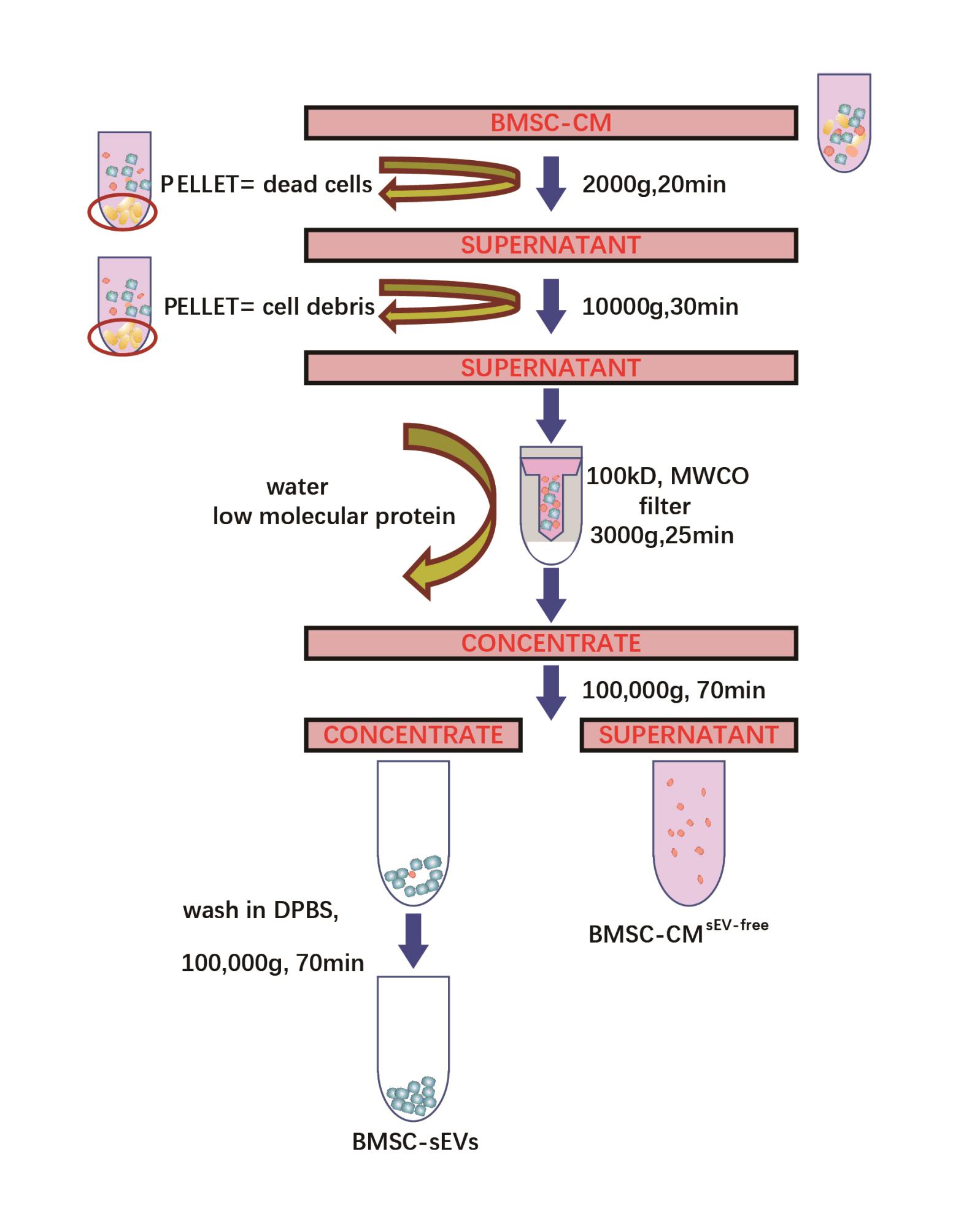


**Fig S1. Protocol for BMSC-sEVs and BMSC-CM^sEV-free^ preparation.**


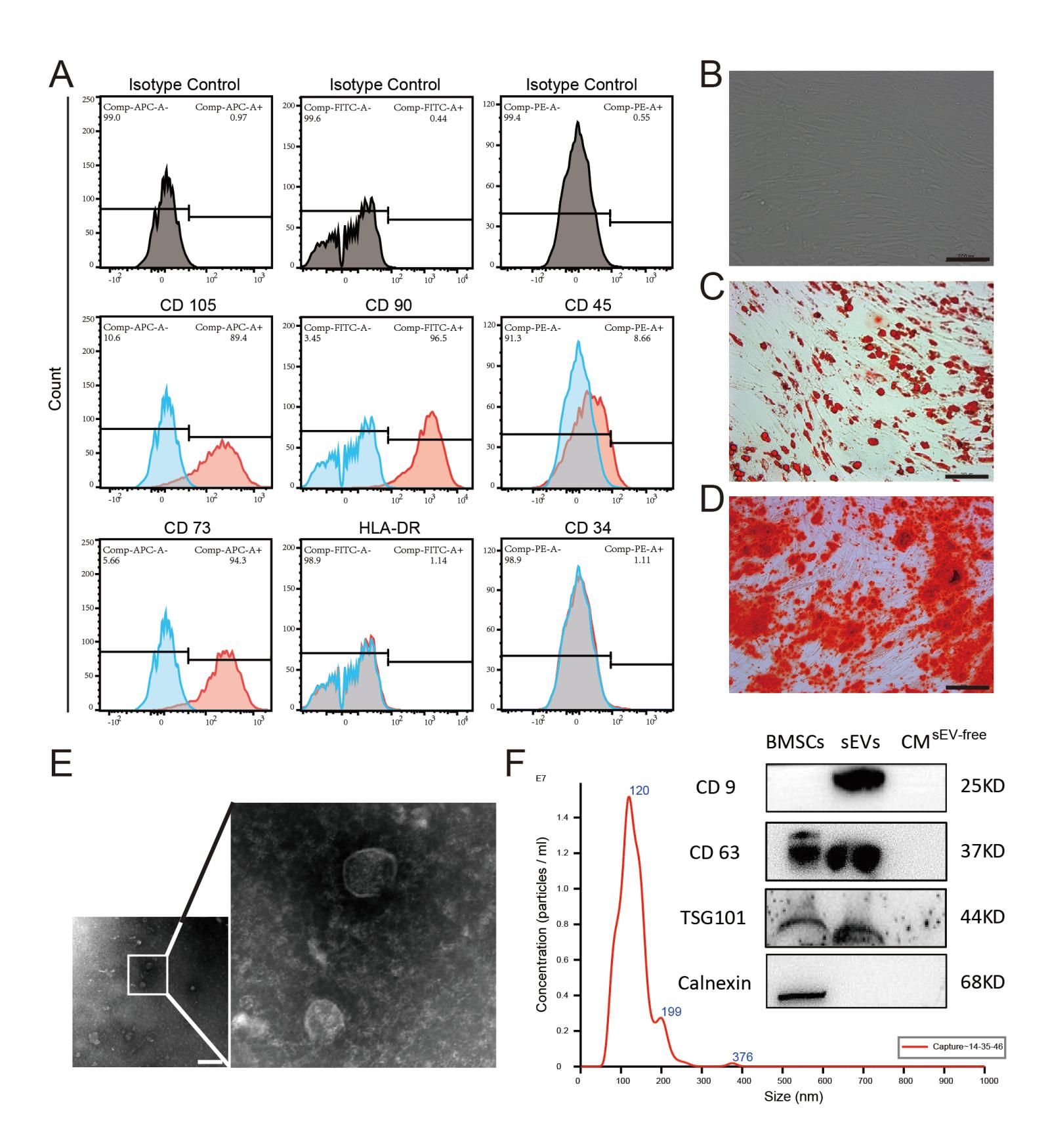


**Fig S2. Characterization of BMSCs and BMSC-sEVs**

The hBMSCs positively expressed CD105, CD90, and CD73 and negatively expressed CD45, CD34, and HLA-DR (Fig. S2A). hBMSCs displayed a spindle-like morphology and were capable of adipogenic and osteogenic differentiation (Fig. S2B-D). Transmission electron microscopy showed that BMSC-sEVs had a cup-shaped double-membrane structure (Fig. S2E). Nanoparticle tracking analysis showed that BMSC-sEVs were mainly distributed at 50–199 nm, with the peak particle size concentrated at 120 nm. BMSC-sEVs expressed CD9, CD63, and TSG101 (Tumor Susceptibility 101) but not calnexin. (Fig. S2F).


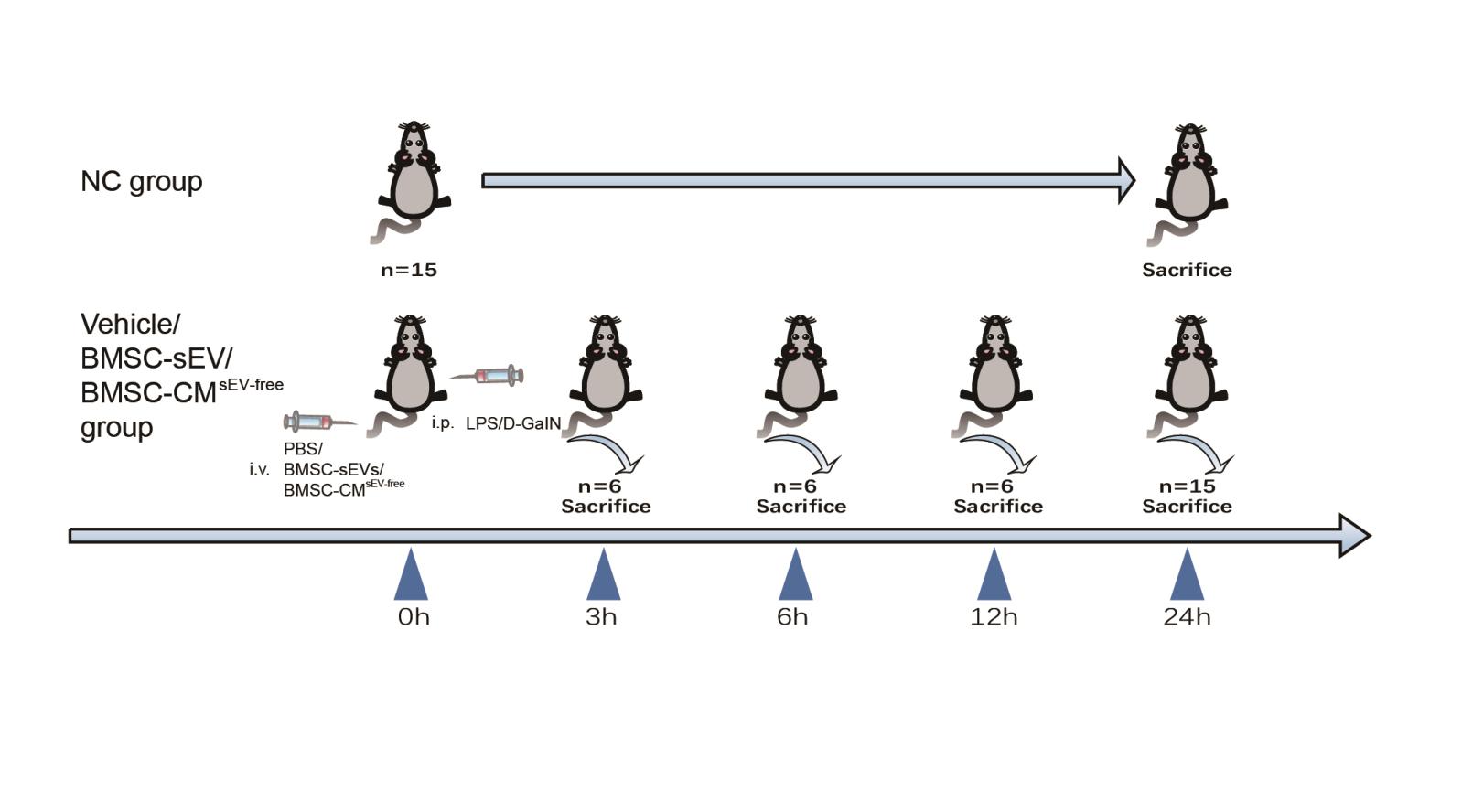


**Fig S3. Experimenta****l protocol for BMSC-sEV treatment of ALF mice.**

**Table.S1 Transfection sequences**

| Name | Sequence (5′–3′) | Length |
| --- | --- | --- |
| mimic NC | Sense UUCUCCGAACGUGUCACGUTT  Antisense ACGUGACACGUUCGGAGAATT | 21  21 |
| miR-20a-5p mimic | Sense UAAAGUGCUUAUAGUGCAGGUAG  Antisense ACCUGCACUAUAAGCACUUUAUU | 23  23 |
| inhibitor NC | CAGUACUUUUGUGUAGUACAA | 21 |
| miR-20a-5p inhibitor | CUACCUGCACUAUAAGCACUUUA | 23 |

**Table.S2 Primer sequences for RT-qPCR**

| Gene | Specie | Primer | Sequence (5′–3′) | Length |
| --- | --- | --- | --- | --- |
| miR-20a-5p | hsa/mmu | sl | GGTTGTTGGTTGGTTGGTTGTATCCAACAACCCTACCT | 38 |
|  |  | F | TGCGTAAAGTGCTTATAGTGC | 21 |
|  |  | R | GTTGTTGGTTGGTTGGTTGT | 20 |
| miR-10a-5p | hsa | sl | GGCTGTTGTGTTGTGTTGTGGATACAACAGCCCACAAA | 38 |
|  |  | F | GGGTACCCTGTAGATCCGAA | 20 |
|  |  | R | GCTGTTGTGTTGTGTTGTGG | 20 |
| miR-22-3p | hsa | sl | GTCCTCCTCTCCTCTCCTCTCATGAGGAGGACACAGTT | 38 |
|  |  | F | GGGAAGCTGCCAGTTGAAG | 19 |
|  |  | R | TCCTCCTCTCCTCTCCTCTC | 20 |
| miR-26b-5p | hsa | sl | GGTGTGGTGTGGTATGGTGTGATCACCACACCACCTAT | 38 |
|  |  | F | AGGGCGTTCAAGTAATTCAGG | 21 |
|  |  | R | GTGTGGTGTGGTATGGTGTG | 20 |
| miR-29a-3p | hsa | sl | GTCCTCCTCTCCTCTCCTCTCATGAGGAGGACTAACCG | 38 |
|  |  | F | AGGGGTAGCACCATCTGAAAT | 21 |
|  |  | R | TCCTCCTCTCCTCTCCTCTC | 20 |
| miR-148a-3p | hsa | sl | GGAGAGGAGAGGAAGAGGGAAATCTCCTCTCCACAAAG | 38 |
|  |  | F | GGGTCAGTGCACTACAGAA | 19 |
|  |  | R | GAGAGGAGAGGAAGAGGGAA | 20 |
| miR-363-3p | hsa | sl | GTCCTCCTCTCCTTCCTTCTCATGAGGAGGACTACAGA | 38 |
|  |  | F | GGGAATTGCACGGTATCCA | 19 |
|  |  | R | TCCTCCTCTCCTTCCTTCTC | 20 |
| U6 | mmu | sl | TCGTATCCATGGCAGGGTCCGAGGTATTCGCCATGGATACGACACAAAAATATGGAACGCTT | 62 |
|  |  | F | GTGCTCGCTTCGGCAGCACA | 20 |
|  |  | R | TGGCAGGGTCCGAGGT | 16 |
| mir-20a pre | mmu | F | CCTGCGTGGTGTGTGTGAT | 19 |
|  |  | R | GGCGAGGCTGGAGTTCTAC | 19 |
| PTEN | hsa | F | AGGGCTTCAATTTCACTTCTT | 21 |
|  |  | R | TTGTACTCCGCTTAAAATCGT | 21 |
| Cyclin D1 | hsa | F | GCGGAGGAGAACAAACAG | 18 |
|  |  | R | CACAGAGGGCAACGAAG | 17 |
| BCL2 | hsa | F | TTCATCGTCCCCTCTCC | 17 |
|  |  | R | TCAGTCCGGTATTCGCA | 17 |
| BAX | hsa | F | TGCGTCCACCAAGAAGC | 17 |
|  |  | R | TCCAGTTCGTCCCCGAT | 17 |
| GAPDH | hsa | F | CCTTCCGTGTCCCCACT | 17 |
|  |  | R | GCCTGCTTCACCACCTTC | 18 |
| Cyclin D1 | mmu | F | ACCCTGACACCAATCTCCT | 19 |
|  |  | R | CTCCTTCTGCACGCACTT | 18 |
| BCL2 | mmu | F | AAACCCTCCATCCTGTCC | 18 |
|  |  | R | TCCTAAACCCTGCTTCCC | 18 |
| BAX | mmu | F | TGCGTCCACCAAGAAGC | 17 |
|  |  | R | CCACCCGGAAGAAGACC | 17 |
| GAPDH | mmu | F | TGTTTCCTCGTCCCGTAGA | 19 |
|  |  | R | ATCTCCACTTTGCCACTGC | 19 |

RT-qPCR, Reverse transcription quantitative polymerase chain reaction; hsa, human; mmu, mouse; pre，precursor; sl, stem loop; F, forward; R, reverse.


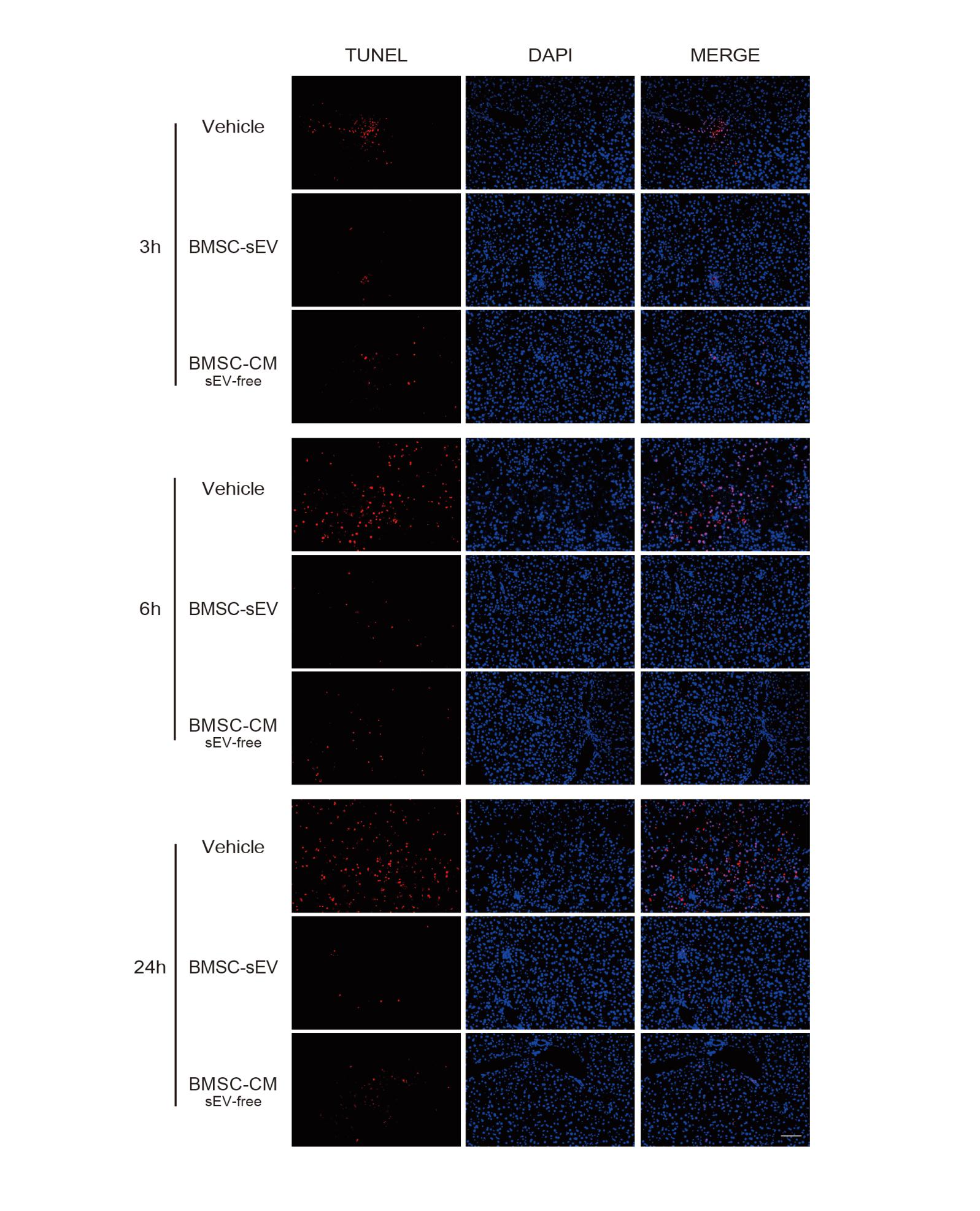


**Fig S4. Representative images of TUNEL staining in liver tissue at 3 h, 6 h and 24h.** Scale bar: 100 μm.

**
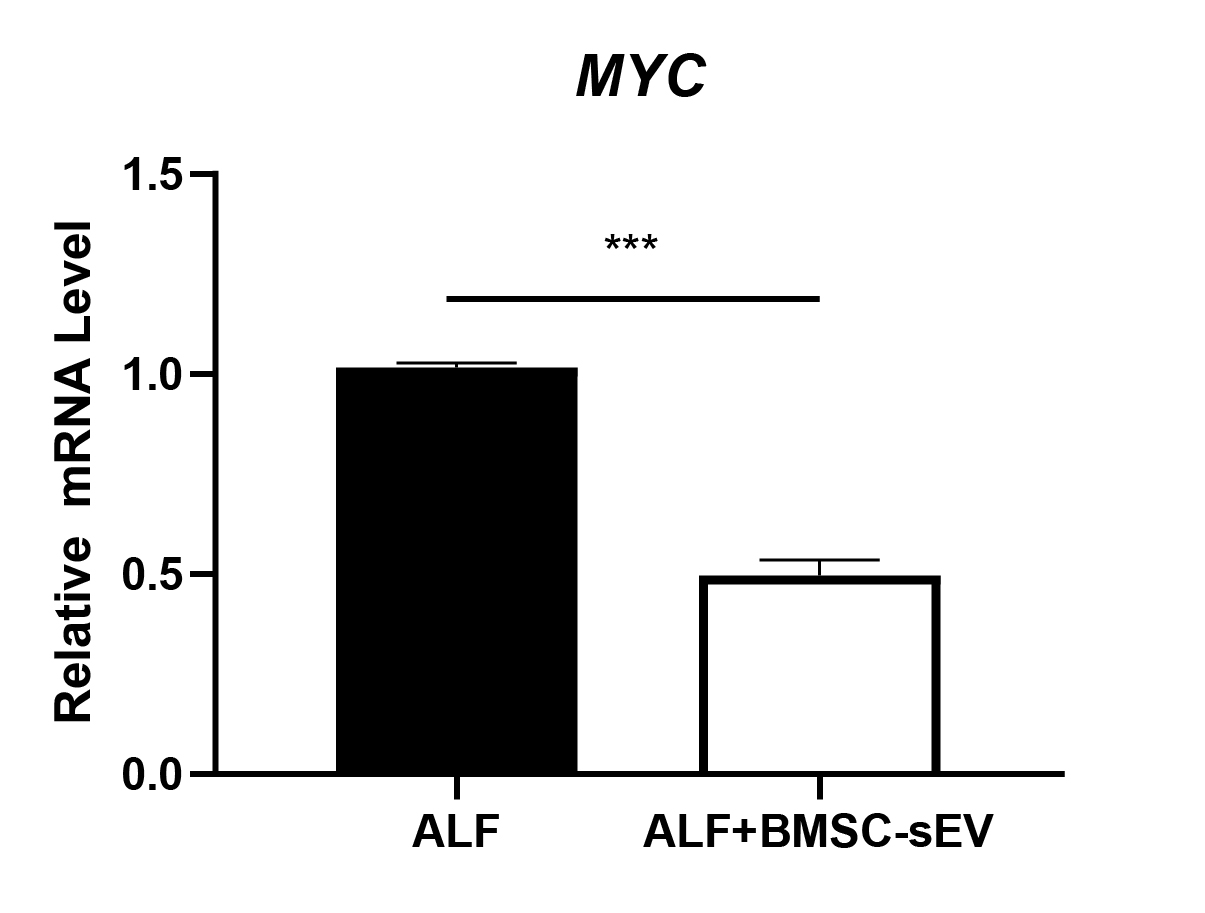
**

**Fig S5. The MYC levels were detected at 24 h after adding BMSC-sEVs to hydrogen peroxide-damaged L02 cells.** n=3. Data are presented as mean ± SEM. Statistical analysis was performed using Student's *t-*test, **p<*0.05, ***p<*0.01*,* ****p<*0.001.

**
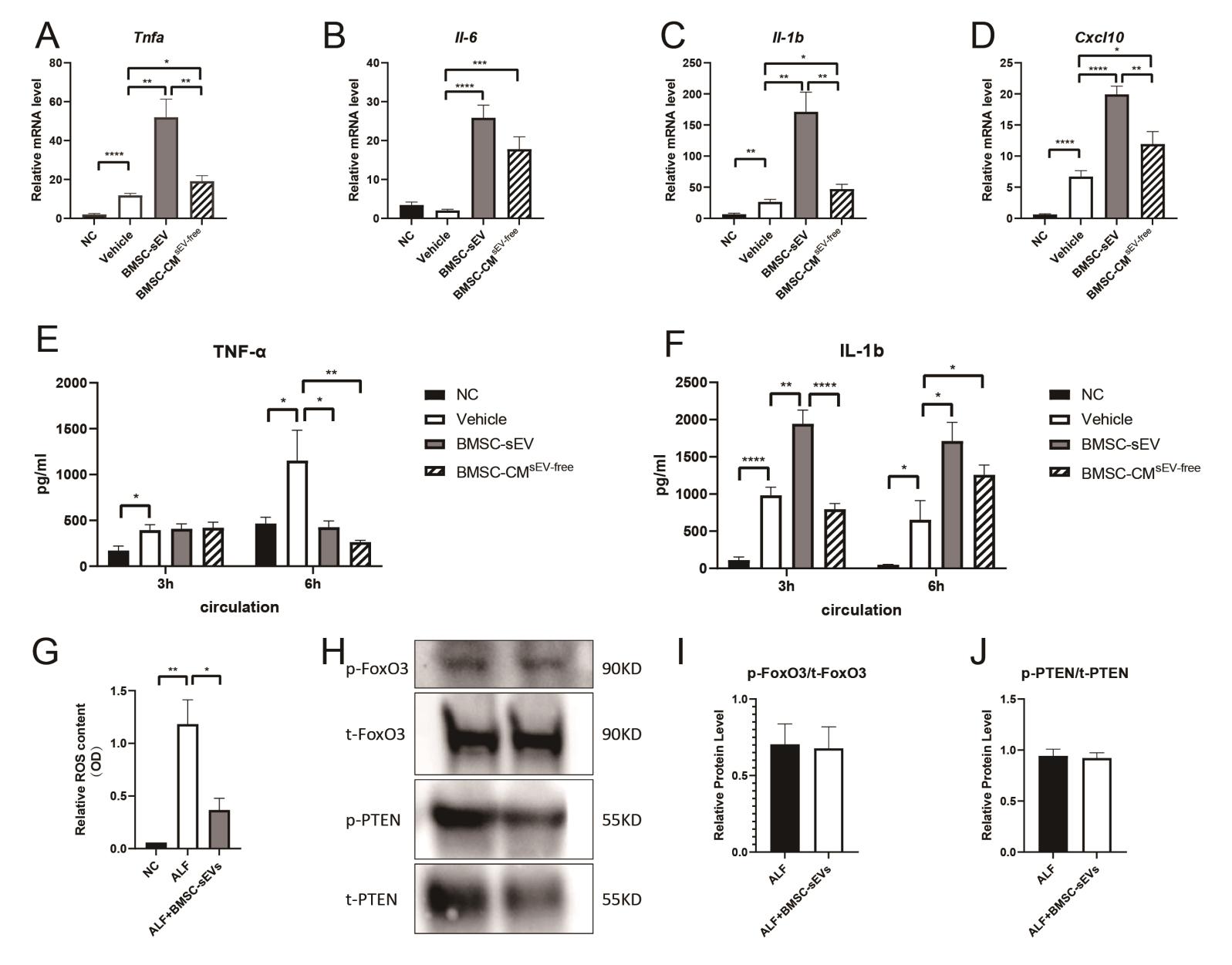
**

**Fig S6. BMSC-sEV promoted the expression of pro-inflammatory and anti-inflammatory factors in the early liver of ALF, and inhibited the oxidative stress of ALF liver cells. (A-D)** qPCR was used to detect the relative expression levels of *Tnfa*, *Il6*, *Il1β* and *Cxcl10* in liver tissues at 3h. n=6. **(E-F)** ELISA was used to detect protein levels of TNF-α and IL-1β in circulation at 3h and 6h. n=6. The ROS**(G)**、 FOXO3A (total and phospho Ser253) and PTEN(total and phospho T366) **(H-J)** were detected at 24 h after adding BMSC-sEVs to hydrogen peroxide-damaged L02 cells. n=3. Data are presented as mean ± SEM. Statistical analysis was performed using Student's *t-*test, **p<*0.05, ***p<*0.01*,* ****p<*0.001.
